# Binding Pattern Reconstructions of FGF-FGFR Budding-Inducing Signaling in Reef-Building Corals

**DOI:** 10.3389/fphys.2021.759370

**Published:** 2022-01-04

**Authors:** Zhuojun Guo, Xin Liao, J.-Y. Chen, Chunpeng He, Zuhong Lu

**Affiliations:** ^1^State Key Laboratory of Bioelectronics, School of Biological Science and Medical Engineering, Southeast University, Nanjing, China; ^2^Guangxi Key Lab of Mangrove Conservation and Utilization, Guangxi Mangrove Research Center, Beihai, China; ^3^Nanjing Institute of Geology and Paleontology, Nanjing, China

**Keywords:** reef-building coral, budding reproduction, receptor tyrosine kinase, full-length transcriptome, FGF-FGFR binding models

## Abstract

Reef-building corals play an important role in marine ecosystems. However, owing to climate change, ocean acidification, and predation by invasive crown-of-thorns starfish, these corals are declining. As marine animals comprise polyps, reproduction by asexual budding is pivotal in scleractinian coral growth. The fibroblast growth factor (FGF) signaling pathway is essential in coral budding morphogenesis. Here, we sequenced the full-length transcriptomes of four common and frequently dominant reef-building corals and screened out the budding-related FGF and FGFR genes. Thereafter, three-dimensional (3D) models of FGF and FGFR proteins as well as FGF-FGFR binding models were reconstructed. Based on our findings, the FGF8-FGFR3 binding models in *Pocillopora damicornis*, *Montipora capricornis*, and *Acropora muricata* are typical receptor tyrosine kinase-signaling pathways that are similar to the Kringelchen (FGFR) in hydra. However, in *P*. *verrucosa*, FGF8 is not the FGFR3 ligand, which is found in other hydrozoan animals, and its FGFR3 must be activated by other tyrosine kinase-type ligands. Overall, this study provides background on the potentially budding propagation signaling pathway activated by the applications of biological agents in reef-building coral culture that could aid in the future restoration of coral reefs.

## Introduction

Coral reefs mainly comprise large numbers of calcium carbonate skeletons produced by scleractinian corals. Coral reefs also serve as a living environment for more than 30% of marine animals and plants (Odum and Odum, 1955; Yu, 2012). Owing to biodiversity and efficient nutrient recycling, coral reefs can affect the physical and ecological conditions of surrounding ocean areas (Connell, 1978). Recently, due to global warming, changes in the physicochemical environment of the ocean, and massive encroachment of the predatory crown-of-thorns starfish (COTS), coral reefs are sharply declining (Moberg and Folke, 1999; Wilson et al., 2006; Nakamura et al., 2014; Reimer et al., 2019; Magel et al., 2020). Currently, scleractinian coral populations are beginning to display features similar to those exhibited during the last mass extinction, such as population shrinkage, the transplanting of colonies to the aphotic zone, and zygote dormancy (Dishon et al., 2020). Thus, determining how to promote the growth activity of reef-building corals is key to coral reef ecological restoration.

The hydra body plan is one of the two basic body plan types in the cnidaria. Anthozoa, including all neontological reef-building corals, have this body type (Kraus et al., 2015; D’Ambra and Lauritano, 2020). Asexual reproduction by budding is a distinctive feature of hydrozoan animals, and is particularly important in maintaining the general skeletal growth of reef-building corals (Otto and Campbell, 1977). Reproduction by budding produces “clones” without going through the embryogenesis stage, and its morphogenesis is controlled by a cascade-inducing signaling pathway (Odum and Odum, 1955). Among these inducing signals, fibroblast growth factors (FGFs) play an important role in budding morphogenesis. The fibroblast growth factor receptor (FGFR) is a typically classic transmembrane dimer receptor activated by FGFs, insulin growth factors (IGFs), and insulin, etc., and belongs to the large class of receptor tyrosine kinases. Tyrosine kinases are enzymes that can transfer a phosphate group from ATP to the tyrosine residues of specific proteins to turn many cellular functions on or off, such as cell proliferation (Weiner and Zagzag, 2000; Lemmon and Schlessinger, 2010; Cadena and Gill, 2015; Ornitz and Itoh, 2015). During bud detachment, FGFR (Kringelchen) is the earliest gene demarcating the parent-bud boundary at the birth site of a new bud (Sudhop et al., 2004; Böttger and Hassel, 2012; Holz et al., 2017; Suryawanshi et al., 2020). FGF signaling molecules are also essential for bud growth as they contribute to tissue development (Lange et al., 2014; Chuang and Mitarai, 2020), including forming the endothelial system, patterning the oral-aboral axis, building the nervous system, etc., (Sudhop et al., 2004; Böttger and Hassel, 2012; Turwankar and Ghaskadbi, 2019). The FGF pathway is conserved at both the amino acid and structural levels. Further, all members of this family share a conserved core region/FGF domain that shows 30–60% sequence similarity (Krishnapati and Ghaskadbi, 2013; Ornitz and Itoh, 2015). Mapping the cell movements and changes in shape during the sprouting process has revealed that FGF and FGFR are used repeatedly to control branch budding and outgrowth (Savage et al., 1993; Tanaka and Gann, 1995; Böttger and Hassel, 2012; Krishnapati and Ghaskadbi, 2013; Tee et al., 2013; Ghaskadbi, 2020). Such findings indicate that from early metazoans to higher vertebrates, FGFs and FGFRs in the budding process are conserved in signaling pathway and functions. Reef-building corals show significant activation of the FGF signaling pathways during induction of polyp bail-out (Wecker et al., 2018; Chuang and Mitarai, 2020). These potential biological functions reveal that budding-inducing signals are crucial to the maintenance of the growth activity of hydrozoan animals. Currently, within the context of population decline of reef-building corals, offering external assistance to budding *via* budding-inducing proteins, such as FGFs, is a potential method for sustaining those populations.

Although reef-building coral transcriptomes have been sequenced by the Illumina platform, issues with short and error splicing triggered by Illumina sequencing can occur, as well as issues caused by individual amplification of target genes, which occurred during the early days of polymerase chain reactions and Sanger sequencing (Rhoads and Au, 2015; Lu et al., 2016; van Dijk et al., 2018). PacBio Sequel II sequencing technology can overcome the limitations of Illumina sequencing technology. To precisely identify the FGF-FGFR binding model in reef-building corals, we sequenced the full-length transcriptomes of four common and frequently dominant reef-building corals, including *Pocillopora damicornis*, *P*. *verrucosa*, *Montipora capricomis*, and *Acropora muricata*, using the PacBio Sequel II platform, screening out related FGFs and FGFRs genes by Nr, Nt, Pfam, KOG, Swiss-Prot, GO, and KEGG annotations, coding sequence predictions, and phylogenetic tree analyses. FGF and FGFR tertiary structures were reconstructed using the trRosetta algorithm and MOE software (Chemical Computing Group Inc., Montreal, Quebec, Canada). FGF-FGFR binding models were reconstructed with the ClusPro v2.0 software package. Illustrating the FGF-FGFR binding models in this manner can guide the generation of biological agents that are used to activate this signaling pathway and promote the budding of reef building corals, ultimately aiding in the recovery of marine ecosystems.

## Materials and Methods

### Ethics

All coral samples were collected and processed in accordance with local laws for invertebrate protection.

### Specimen Collection

The species in our study were collected from the Xisha Islands in the South China Sea (latitude 15°40′–17°10′ north, longitude 111°–113° east). All samples collected in this study were retrieved from the newly budded branches.

### Coral Culture System

We used three sample replicates from the same newly budded branch for library construction and sequencing. The coral samples were cultured in our laboratory coral tank with conditions conforming to the environment of their habitat. All species were raised in a RedSea^®^ tank (redsea575, Red Sea Aquatics Ltd.) at 26C and 1.025 salinity (Red Sea Aquatics Ltd.). The physical conditions of the coral culture system are as follows: three coral lamps (AI^®^, Red Sea Aquatics Ltd.), a protein skimmer (regal250s, Reef Octopus), a water chiller (tk1000, TECO Ltd.), two wave devices (VorTechTM MP40, EcoTech Marine Ltd.), and a calcium reactor (Calreact 200, Reef Octopus).

### RNA Extraction

All RNA extraction procedures were carried out according to the manufacturer’s instructions. Total RNA was isolated with TRIzol LS Reagent (Thermo Fisher Scientific, 10296028) and treated with DNase I (Thermo Fisher Scientific, 18068015). High-quality mRNA was isolated with a FastTrack MAG Maxi mRNA Isolation Kit (Thermo Fisher Scientific, K1580-02). Samples were separated from healthy *P*. *damicornis*, *P*. *verrucosa*, *M*. *capricomis*, and *A*. *muricata* to ensure that enough high-quality RNA (>10 μg) could be obtained for a full-length cDNA transcriptome library.

### Library Construction

Before establishing the library, the quality of the total RNA was determined. Agarose gel electrophoresis was used to analyze the degree of degradation of RNA and possible contamination. A Nanodrop nucleic acid quantifier was used to detect the purity of RNA (OD260/280 ratio), a Qubit RNA assay was used to accurately quantify the RNA concentration, and an Agilent 2200 TapeStation was used to accurately detect the integrity of the RNA. The Clontech SMARTer^®^ PCR cDNA Synthesis Kit (Clontech Laboratories, 634926) and the BluePippin Size Selection System protocol, as described by Pacific Biosciences (PN 100-092-800-03), were used to prepare the Iso-Seq library according to the Isoform Sequencing protocol (Iso-Seq).

### Sequencing and Data Processing

We used the PacBio Sequel II platform with single molecular real time (SMRT) sequencing technology and SMRTlink v7.0 software (minLength 50; maxLength 15,000; minPasses 1) to process sequencing samples. After polymer read bases were performed (Chin et al., 2016), the subreads.bam files were obtained by removing the joint and the original offline data, where the length was less than 50 bp. The circular consensus sequences (CCSs) were obtained using the subreads.bam file through the CCS algorithm, which is self-correcting for single molecule multiple sequencing. Consequently, the full-length-non-chimera (FLNC) and non-full-length (nFL; non-chimera) sequences were identified by determining whether CCSs contained 5′-primer, 3′-primer, and poly-A. FLNC sequences of the same transcript were clustered by a hierarchical n * log (n) algorithm to obtain consensus sequences. The corrected consensus reads were polished from consensus sequences (Arrow polishing) using LoRDEC v0.7 software and the RNA-seq data sequenced by the Illumina HiSeq X Ten platform (Salmela and Rivals, 2014). Using CD-HIT software (-c 0.95 -T 6 -G 0 -aL 0.00 -aS 0.99), all redundancies were removed in corrected consensus reads to acquire final full-length transcripts and unigenes for subsequent bioinformatics analysis (Fu et al., 2012).

### Gene Functional Annotation

Gene function was annotated using the following databases: Nr (NCBI non-redundant protein sequences) (Li et al., 2002), Nt (NCBI non-redundant nucleotide sequences), Pfam (Protein family), KOG (Clusters of Orthologous Groups of proteins) (Tatusov et al., 2003), Swiss-Prot (a manually annotated and reviewed protein sequence database) (Bairoch and Apweiler, 2000), GO (Gene Ontology) (Ashburner et al., 2000), and KEGG (Kyoto Encyclopedia of Genes and Genomes) (Kanehisa et al., 2004). We use BLAST 2.7.1+ (Altschul et al., 1990) in NCBI to set the *e*-value “1e−5” for Nt database analysis; Diamond v0.8.36 BLASTX software to set the *e*-value to “1e−5” for Nr, KOG, Swiss-Prot, and KEGG database analyses; and the HMMER 3.1 package for Pfam database analysis.

### Coding Sequence Analysis

Coding sequences were predicted by ANGEL v2.4 software in fault-tolerant mode, which maximizes the limited information from the input sequence to predict the coding sequence (Shimizu et al., 2006).

### Phylogenetic Analysis

The amino acid sequences were constructed into phylogenetic trees using MEGA X software by the neighbor-joining (NJ) method (Saitou and Nei, 1987; Kumar et al., 2018). The evolutionary history of the analyzed taxa is represented by the bootstrap consensus tree drawn from 1,000 replicates (Felsenstein, 1985). The percentage of replicate trees next to the branches in which the associated taxa are together is presented. The Poisson correction method was used to compute the evolutionary distances in units of the number of amino acid substitutions per site (Zuckerkandl and Pauling, 1965). A matrix of pairwise distances was estimated by using the JTT model and then selecting the topology with the highest log likelihood value.

### Homological Gene Selection

To precisely construct the phylogenetic trees, the FGF8 protein sequences of *P*. *damicornis* (Pd_FGF8), *P*. *verrucosa* (Pv_FGF8), *M*. *capricomis* (Mc_FGF8), *A*. *muricata* (Am_FGF8), *Hydra vulgaris* (XP_012554564.1), *Orbicella faveolata* (XP_020606946.1), *A*. *millepora* (XP_029189212.1), *A*. *digitifera* (XP_015756877.1), *Nematostella vectensis* (XP_032240538.1), *Actinia tenebrosa* (XP_031555139.1), *Stylophora pistillata* (XP_022781642.1), *Exaiptasia diaphana* (XP_020913607.1), *Denticeps clupeoides* (XP_028829118.1), *Trematomus bernacchii* (XP_033987865.1), *Kryptolebias marmoratus* (XP_017277162.1), *Astatotilapia calliptera* (XP_026046852.1), *Toxotes jaculatrix* (XP_040905908.1), *Melanotaenia boesemani* (XP_041865608.1), *Perca flavescens* (XP_028459575.1), *Etheostoma cragini* (XP_034754871.1), *E*. *spectabile* (XP_032367422.1), *Perca fluviatilis* (XP_039639039.1), *Cynoglossus semilaevis* (XP_016892646.1), *Sander lucioperca* (XP_016892646.1), *Maylandia zebra* (XP_004573854.1), *Cottoperca gobio* (XP_029305840.1), *Carcharodon carcharias* (XP_041065901.1), *Scyliorhinus canicular* (XP_038678019.1) and *Rhincodon typus* (XP_020371706.1) were selected and the FGFR protein sequences of *P*. *damicornis* (Pd_FGFR3), *P*. *verrucosa* (Pv_FGFR3), *M*. *capricomis* (Mc_FGFR3) *A*. *muricata* (Am_FGFR3), *H*. *vulgaris* (NP_001296694.1), *S*. *pistillata* (XP_022781630.1), *A*. *millepora* (XP_029189174.1), *A*. *digitifera* (XP_015756845.1), *N*. *vectensis* (XP_032231385.1), *E*. *diaphana* (KXJ23083.1), and *O*. *faveolata* (XP_020606906.1) were selected by setting the *e*-value threshold in BLAST to 1e−10 and then selecting FGFR sequences of which the species are Cnidarians and FGF sequences where species present an *e*-value lower than 1e−10.

### Prediction of the Protein Tertiary Structure

The FGF8 tertiary structures were predicted using the trRosetta algorithm (Yang et al., 2020). trRosetta is an algorithm for fast and accurate *de novo* protein structure prediction that builds the protein structure based on direct energy minimization with a restrained Rosetta. The restraints include inter-residue distance and orientation distributions, predicted by a deep residual neural network.

The template crystal FGFR3 structures were identified through BLAST (Camacho et al., 2009) and downloaded from the RCSB Protein Data Bank (PDB ID: 6PNX for Pd_FGFR3, Mc_FGFR3 and Am_FGFR3, 4ZSA for Pv_FGFR3). Homology modeling was conducted in MOE (Maier and Labute, 2014; Kandathil et al., 2019; Molecular Operating Environment [Moe], 2019). The protonation state of the protein and the orientation of the hydrogens were optimized by LigX at a pH of 7.0 and temperature of 26.85C. First, the target sequence was aligned to the template sequence, and ten independent intermediate models were built. These different homology models were the result of the permutational selection of different loop candidates and side chain rotamers. Thereafter, the intermediate model that scored the best, according to the GB/VI scoring function, was selected as the final model, and subjected to further energy minimization using the AMBER12/EHT force field.

### Molecular Docking

Protein-protein docking with the ClusPro server (Kozakov et al., 2017) was used for molecular docking simulations of four complexes: Pd_FGFR3 with Pd_FGF8, Pv_FGFR3 with Pv_FGF8, Mc_FGFR3 with Mc_FGF8, and Am_FGFR3 with Am_FGF8. For protein docking, the smaller protein (a smaller number of residues) is often set as the ligand and the larger protein is often set as the receptor. The ligand was rotated 70,000 times. For each rotation, the ligand was translated in the x, y, and z axes relative to the receptor on a grid. One translation with the best score was selected from each rotation. Of the 70,000 rotations, 1,000 rotation/translation combinations that had the lowest scores were selected. Thereafter, a greedy clustering of these 1,000 ligand positions with a 9 Å C-alpha root mean squared deviation (RMSD) radius was performed to identify the ligand positions with the most “neighbors” within 9 Å (i.e., cluster centers). The top ten cluster centers with the most cluster members were then retrieved and individually inspected visually. The intermolecular contacts from the most probable position were further evaluated.

## Results

### Full-Length Transcriptome Sequencing and Data Processing of Four Species of Coral

The SMRT-sequencing was performed with the PacBio Sequel II platform to acquire offline polymer read bases of full-length transcriptomes using SMRTlink v7.0 software (Methods). The offline polymer read bases of *P*. *damicornis*, *P*. *verrucosa*, *M*. *capricomis*, and *A*. *muricata* samples were 44.33G, 42.67G, 41.63G, and 27.8G, respectively. The polymer read bases, subreads, CCSs, FLNCs, consensus sequences, corrected consensus reads, and unigenes are shown in Table 1, which also contains the information revealed in subsequent analyses.

**TABLE 1:** Sequencing data statistics of four full-length coral transcriptomes.

Sample name	*P. damicornis*	*P. verrucosa*	*M. capricomis*	*A. muricata*
Polymerase read base (G)	44.33	27.8	41.63	42.67
Subread base (G)	42.84	26.52	40.29	41.56
Subread (number)	19,729,634	16,171,363	17,878,457	14,983,676
Average subread length (Nt)	2,172	1,641	2,254	2,774
CCS (number)	602,185	292,565	634,369	646,298
FLNC (number)	463,766	249,577	452,189	496,518
Consensus read (number)	38,663	24,860	35,293	41,489
Corrected consensus (number)	38,663	24,860	35,293	41,489
Unigene (number)	22,408	13,173	20,263	23,499

The full-length transcriptomes were annotated with Nr, Nt, Pfam, KOG, Swiss-Prot, GO, and KEGG databases, and the related unigene statistics are shown in Table 2. In protein-related databases, approximately 90% of the unigenes of investigated corals are annotated in Nr, which is the basic protein primary sequence database. Over 60% of the unigenes are annotated in Pfam, a conserved domain database. Over 70% of the unigenes are annotated in Swiss-Prot, a manually screened protein sequence database. As shown in Supplementary Figures 1–4, the Nr unigene annotations revealed that the genes of the four investigated corals are closest to the cnidarians *A*. *digitifera*, *Exaiptasia pallida*, and *N*. *vectensis*, with over 80% unigene overlap. Such findings indicate the accuracy and credibility of the annotation results. The coding sequences (CDS) were analyzed with ANGEL v2.4 software, and the ANGEL.pep files of the protein profiles were subsequently obtained (Supplementary Material 2) (Methods). Based on the Nr results and ANGEL.pep files, the FGF-FGFR budding inducing signals were identified in the four corals studied (Methods).

**TABLE 2:** Annotation result statistics of four full-length coral transcriptomes.

Sample name	*P. damicornis*	*P. verrucosa*	*M. capricomis*	*A. muricata*
NR (number)	20,749	11,787	19,149	22,668
NT (number)	6,052	3,299	13,562	22,826
KOG (number)	13,861	7,749	12,832	15,138
Swiss-Prot (number)	16,759	9,360	15,492	18,466
GO (number)	15,456	8,380	14,033	16,546
Pfam (number)	15,456	8,380	14,033	16,546
KEGG (number)	19,721	11,069	18,191	21,515

### Phylogenetic Analysis of FGF-FGFR Budding Inducing Signals

**FIGURE 1:**
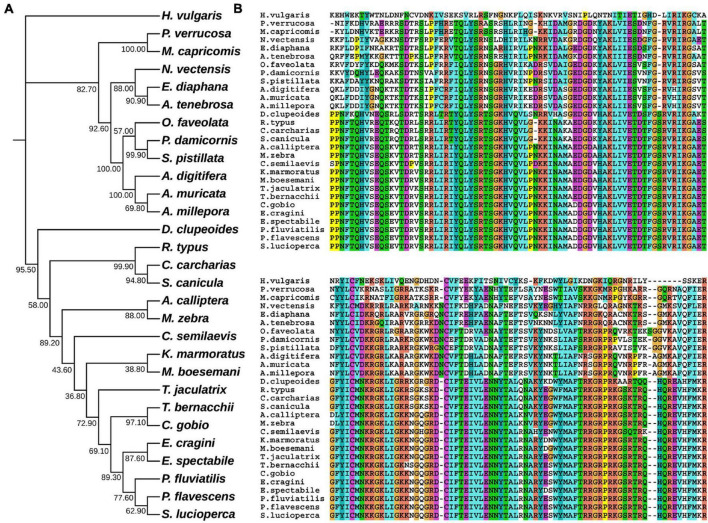
Evolutionary phylogenetic tree of FGF8s. **(A)** Bootstrap consensus tree reconstructed with MEGA X using neighbor-joining with default settings. The values beside the branches represent the percentage of time that a node was supported over 1,000 bootstrap replications. **(B)** Partially conserved domains of FGF genes.

**FIGURE 2:**
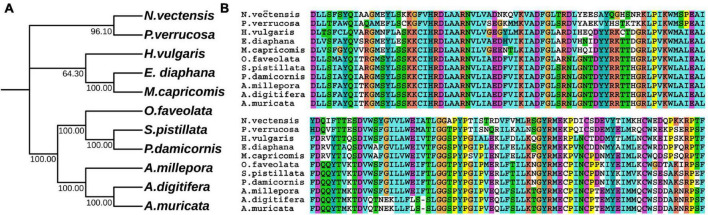
Evolutionary phylogenetic tree of FGFR3s. **(A)** Bootstrap consensus tree reconstructed with MEGAX using neighbor-joining with default settings. The values beside the branches represent the percentage of time that a node was supported over 1,000 bootstrap replications. **(B)** Partially conserved domains of FGFR3 genes.

In hydrozoan animals previously studied, the receptor tyrosine kinase related to the induction of polyp budding morphogenesis is FGFR3 (Kringelchen), and its general ligand is FGF8/17/18. To explore the evolutionarily homologous protein sequences of FGF and FGFR in four corals, phylogenetic trees were constructed using MEGA X based on multiple sequence alignments, according to the FGF8 and FGFR3 protein sequence results of four corals in the ANGEL.pep files and NCBI database (Figures 1, 2). Based on the phylogenetic trees, both FGF8 and FGFR3 are widely distributed in hydrozoan animals, and had evolved from the last common ancestor with the primary hydra body plan. After phylogenetic analysis, the FGF8 and FGFR3 protein sequences of the four corals were precisely determined, namely: Pd_FGF8, Pv_FGF8, Mc_FGF8, Am_FGF8, Pd_FGFR3, Pv_FGFR3, Mc_FGFR3, and Am_FGFR3 (Supplementary Data). Based on these definitive protein primary sequences, 3D modeling reconstructions were performed.

### 3D Modeling Reconstructions of FGF8 and FGFR3

**FIGURE 3:**
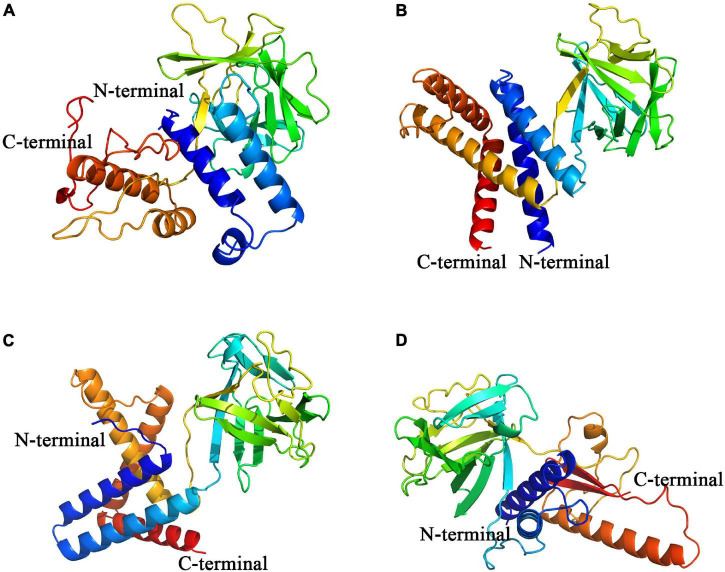
Constructed *de novo* models of coral FGF8 proteins. **(A–D)** Are *de novo* FGF8 models of *P. damicornis*, *P. verrucosa*, *M. capricomis* and *A. muricata*, respectively. C-terminals are marked in red and N-terminals are marked in blue.

The modeling results of Pd_FGF8, Pv_FGF8, Mc_FGF8, and Am_ FGF8 are depicted in Figure 3 and Supplementary Figure 5. Protein structures revealed standard FGF features (Figure 3) while Ramachandran plots demonstrated that 99% of the residues exist in allowed regions, indicating that the 3D structures of the FGF8 model are reasonable (Supplementary Figure 5). Pd_FGF8 has one main beta strand region and four alpha helix regions (Figure 3A), while Pv_FGF8 has one main beta strand region and five alpha helix regions (Figure 3B). Further, the structure of Mc_FGF8 has one main beta strand region and five alpha helix regions (Figure 3C), while Am_ FGF8 has two main beta strand regions and two alpha helix regions (Figure 3D).

**FIGURE 4:**
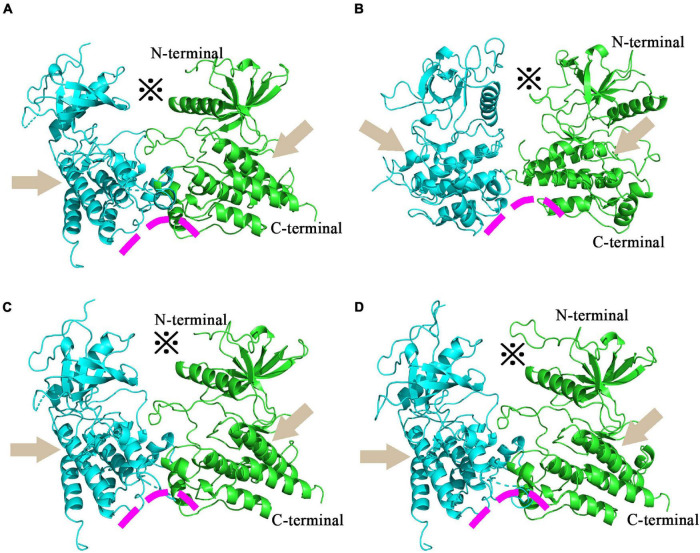
Homology models of coral FGFR3 dimers. Homology models of *P*. *damicorni*, *P*. *verrucosa*, *M*. *capricomis* and *A*. *muricata* FGFR3 dimers are shown in panels **(A–D)**. These reconstructions illustrate that the FGFR3s of the four corals are all classic receptor tyrosine kinases with standard molecular architectural features, including extracellular ligand-binding domains (※), transmembrane helixes (arrows), and juxta-membrane regulatory regions (arcs).

**FIGURE 5:**
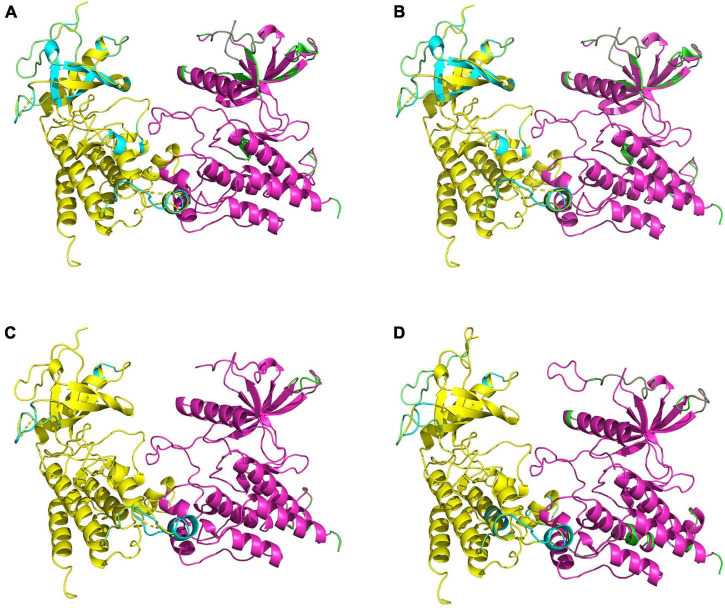
Comparisons of homology-constructed coral FGFR3 dimers with receptor tyrosine kinase templates from the RCSB Protein Data Bank. Superposition results of FGFR3 dimer model structures and related template structures in *P. damicorni*, *P. verrucosa*, *M. capricomis*, and *A. muricata* are shown in panels **(A–D)**. The FGFR3 dimer structure is shown in yellow and purple, and the template structure is shown in green and cyan. The high degree of overlap indicates that coral FGFR3 dimers and receptor tyrosine kinase templates are highly coincident, confirming that the four coral FGFR3s are all tyrosine kinase receptors.

**FIGURE 6:**
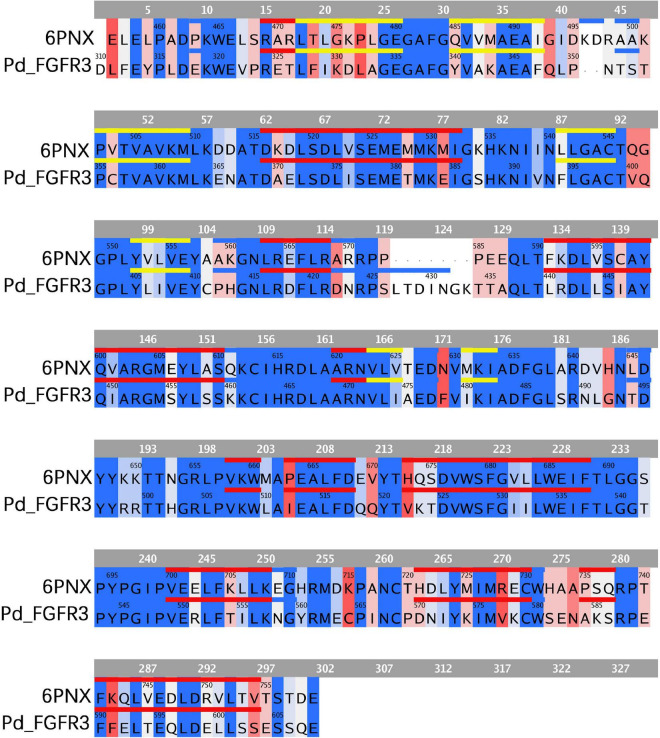
Sequence comparison between FGFR3 in *P*. *damicornis* and its template. The same or similar residues are highlighted in blue and dissimilar ones are highlighted in red, with darker blue indicating more similar residues and darker red indicating more dissimilar residues. The sequences corresponding to alpha helixes and beta strands are marked with red and yellow lines, respectively. The FGFR3 dimer structure is basically consistent with the template structure.

Fibroblast growth factor receptors, including FGFR3, exist as a type of cellular transmembrane dimer. Dimer modeling results for Pd_FGFR3, Pv_FGFR3, Mc_FGFR3, and Am_FGFR3 are depicted in Figure 4 and Supplementary Figure 6. Figure 4 illustrates that the FGFRs of the four investigated reef-building corals are a type of classic receptor tyrosine kinase. Further, Ramachandran plots for FGFR3s revealed that 99% of the residues exist in allowed regions (Supplementary Figure 6). Structural analyses of the FGFR3 dimer modeling results are shown in Figures 5, 6 and Supplementary Figures 7–9. The average RMSD values of the 3D structures overlapping with template structures for Pd_FGFR3, Pv_FGFR3, Mc_FGFR3, and Am_FGFR3 are 0.323Å, 0.214Å, 0.116Å, and 0.260Å. Both constructed dimer models and their template structures were found to have the same alpha helix and beta strand regions (Figure 5). The overall identities of the amino acid sequences for Pd_FGFR3, Pv_FGFR3, Mc_FGFR3, and Am_FGFR3 are 59.08, 36.57, 61.94, and 54.13% by BLAST, respectively (Figure 6 and Supplementary Figures 7–9).

### Binding Modes of Coral FGF8 and FGFR3

**FIGURE 7:**
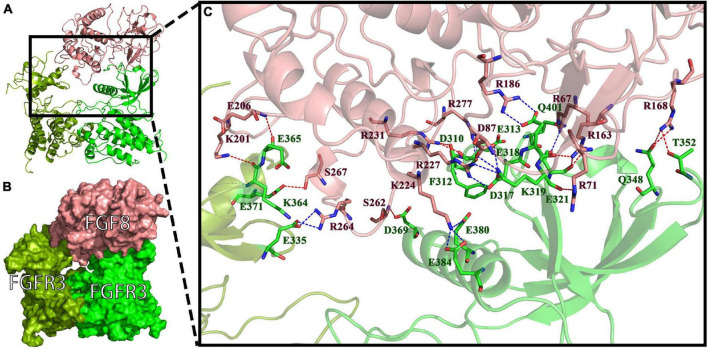
FGF8-FGFR3 binding pattern docked by ClusPro in *P*. *damicornis*. **(A)** The interaction between Pd_FGF8 and Pd_FGFR3. **(B)** The surface binding model of Pd_FGF8 and Pd_FGFR3. Pd_FGFR3 chain A is bright green, Pd_FGFR3 chain B is colored pea green and Pd_FGF8 is colored pink. **(C)** Details of the interaction between Pd_FGF8 and Pd_FGFR3. The residues in Pd_FGFR3 are green, and in Pd_FGF8 are pink. The red dashes represent hydrogen bond interactions and the blue dashes represent salt bridges.

To investigate the binding mode of FGF8s and FGFR3s, docking simulation studies were carried out. The interaction between Pd_FGFR3 and Pd_FGF8 is shown in Figures 7A,B. The contact list between Pd_FGFR3 and Pd_FGF8 is shown in Figure 7C and Supplementary Table 1. Docking simulation studies indicate that amino acid residues of D310, F312, E313, D317, E318, K319, E321, Q348, T352, D369, E380, E384, and Q401 in chain A, and E335, K364, E365, and E371 in chain B bind with R67, R71, D87, R163, R168, R186, K201, E206, K224, R227, R231, S262, R264, S267, and R277 in Pd_FGF8 through salt bridges and hydrogen bond interactions (Figure 7C). A total of 279 residues were found in Pd_FGF8, of which fifteen interact with the Pd_FGFR3 dimer. Of these fifteen residues, Arg163, Arg168, Arg231, Arg67, and Lys224 interact with Glu318, Glu321, Thr352, Gln348, Phe312, Glu313, Gln401, Glu321, Glu380, and Glu384 in Pd_FGFR3 chain A by one binding to two models. Arg186, Arg227, Arg277, and Ser262 interact with Glu318, Asp317, Asp310, and Asp369 in Pd_FGFR3 chain A by one binding to one model. Arg71 and Asp87 simultaneously interact with Lys319 in Pd_FGFR3 chain A. Arg264, Glu206, Lys201, and Ser267 interact with Glu335, Glu365, Lys364, and Glu371 in Pd_FGFR3 chain B by one binding to one model (Supplementary Table 1).

**FIGURE 8:**
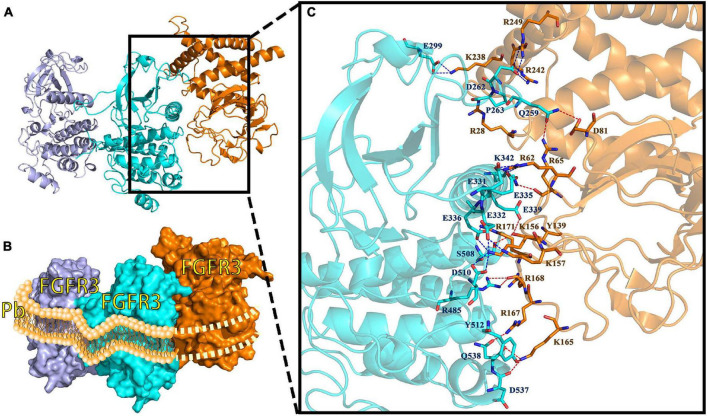
FGF8-FGFR3 binding pattern docked by ClusPro in *P*. *verrucosa*. **(A)** The interaction between Pv_FGF8 and Pv_FGFR3. **(B)** Surface binding model of Pv_FGF8 and Pv_FGFR3. Pd_FGFR3 chain A is colored lightpurple, Pd-FGFR3 chain B is colored aquamarine, and Pd_FGF8 is orange. **(C)** Details of the interaction between Pv_FGF8 and Pv_FGFR3. The residues in Pv_FGFR3 are cyan, and in Pv_FGF8 they are orange. The red dashes represent hydrogen bond interactions and the blue dashes represent salt bridges. Interaction sites between FGFR3 and FGF8 occur only in FGFR3 chain A, which simultaneously falls into the lipid bilayer of the cell membrane, a situation that does not make sense. Pb, phospholipid bilayer of cell membrane.

The interaction between Pv_FGFR3 and Pv_FGF8 is shown in Figures 8A,B. The contact list between Pv_FGFR3 and Pv_FGF8 is shown in Supplementary Table 2. Docking simulation studies indicate that the amino acid residues of Q259, D262, P263, E299, E331, E332, E335, E336, E339, K342, R485, S508, D510, Y512, D537, and Q538 in chain A bind with R28, R62, R65, D81, Y139, K156, K157, K165, R167, R168, R171, K238, R242, and R249 in Pv_FGF8 through salt bridges and hydrogen bond interactions (Figure 8C). FGF8 only connects to residues in FGFR chain A and has no chemical connection to chain B.

The interaction between Mc_FGFR3 and Mc_FGF8 is shown in Supplementary Figures 10A,B. The contact list between Mc_FGFR3 and Mc_FGF8 is shown in Supplementary Table 3. Docking simulation studies indicate that the amino acid residues of E487, E489, D545, and R669 in chain A, and E487, R500, R516, L539, H546, D550, Y668, K670, and R675 in chain B bind with M1, Y12, Q24, Q29, R44, A211, K212, E214, D215, K218, D219, Y230, K231, R234, Q235, and R242 in Mc_FGF8 through salt bridges and hydrogen bond interactions (Supplementary Figure 10C). FGF8 is linked to FGFR chain A by eight residues, one of which is linked to two residues of FGFR chain A on position 234 of the residue (Arg234) and FGFR chain B by eleven residues. The 24th residue (Gln24) in FGF is linked to the 31st residue and the 54th residue of FGFR chain B. FGFR is also connected with two or more residues in FGF through three residues in chain A and three residues in chain B to further strengthen the structural stability of the receptor ligand.

The interaction between Am_FGFR3 and Am_FGF8 is depicted in Supplementary Figures 11A,B. The contact list between Am_FGFR3 and Am_FGF8 is shown in Supplementary Table 4. The docking simulation studies indicate that the amino acid residues of Y301, D303, and D307 in chain A, and T300, D303, D304, E360, S363, D364, and E366 in chain B bind with R162, R185, Y192, K193, R195, R196, K198, R212, R213, K214, T216, Y217, and L274 in Am_FGF8 through salt bridges and hydrogen bond interactions (Supplementary Figure 11C). The extracellular ligand-binding domains in Am_FGFR3 interact with Am_FGF8 through three residues in chain A and eight residues in chain B.

In summary, our research suggests that the FGF8-FGFR3 binding patterns in *A*. *muricata*, *P*. *damicornis*, and *M*. *capricomis* are typical of a receptor tyrosine kinase signaling pathway, with one FGF binding to an FGFR dimer in the active-binding region of extracellular ligand-binding domains. However, in *P. verrucosa*, the reconstruction result revealed that Pv_FGF8 only interacts with Pv_FGFR3 chain A, which is found in the middle of the lipid bilayer of the cell membrane, which is inexplicable based on real cellular processes. Such finding indicates that Pv_FGF8 is not the ligand of Pv_FGFR3; thus, it must be activated by other ligands.

## Discussion

Currently, the full-length transcriptome of marine organisms acquired by PacBio Sequel II sequencing technology enables researchers to directly obtain *de novo* completed unigene profile, including intact 5′UTR and 3′UTR in a more high-efficiency manner (Cheng et al., 2019). The provided data allow in-depth biological research. Further, bioinformatics researchers can also directly extract interesting gene data for phylogenetic studies. With these tools, researchers can accurately and more efficiently analyze all gene expression profile information, such as gene expression, variable splicing, gene fusion, expression regulation, CDS, and protein structure, overcoming the limitations and problems of next-generation sequencing technology (Rhoads and Au, 2015; Lu et al., 2016; van Dijk et al., 2018).

This study revealed the full-length transcriptomes and protein CDS profiles of four dominant reef-building corals. The goal of this computational study was to reconstruct the binding conformations and interactions between FGFs and FGFRs, and determine key FGF-FGFR binding patterns of reef-building corals. Based on full-length protein sequences, the results of multiple sequence alignment show that FGF and FGFR proteins in hydrozoan animals share sequence segments and features. Ramachandran plots of FGFR3s show that 99% of the residues exist in allowed regions and the RMSD values of FGFR3s are fairly low, indicating that all 3D model structures are reasonable (Figure 6 and Supplementary Figures 5–9). 3D reconstructions of the FGFR dimers revealed the entire N-terminal amino acid residues exposed in the extracellular region, which was sufficient for calculating binding sites with ligands (Tavormina et al., 1995; Gupte et al., 2011). The N-terminal domain of receptor tyrosine kinase is composed of stranded β-sheets and α-helixes, and the C-terminal domain is a large cytoplasmic domain with α-helixes (Mohammadi et al., 1996; Linger et al., 2008; Trenker and Jura, 2020). As a typical receptor tyrosine kinase, the FGFR dimer has alpha-helical transmembrane domains and juxta-membrane regulatory regions found at sites where dimer structures are connected (Iwamoto et al., 2005). In our study, all reconstructed 3D FGFR3 dimers from four reef-building corals had molecular architecture features typical of tyrosine kinase receptors, including an extracellular ligand-binding domain, transmembrane helixes, and juxta-membrane regulatory regions (Figures 4, 5).

Receptor tyrosine kinases become activated through autophosphorylation, which is thought to be induced through the mechanism of ligand-mediated receptor oligomerization (Ullrich and Schlessinger, 1990). Receptor activation results in a signal transduction cascade that leads to gene activation and diverse biological responses (Johnson and Williams, 1993; Naski and Ornitz, 1998). Because the FGF8-FGFR3 binding pattern in *A*. *muricata*, *P*. *damicornis*, and *M*. *capricomis* belong to the pattern of a dimeric assemblage of one ligand and one receptor dimer, this type of binding pattern might be an evolutionary ancestral feature of reef-building corals inherited by most species (Figures 7, 8 and Supplementary Figures 10, 11; Johnson and Williams, 1993; Stauber et al., 2000).

In this study, we sequenced and annotated the full-length transcriptomes of four common and frequently dominant reef-building corals, reconstructed their FGFR3 receptor tyrosine kinases, and found that FGF8 is the ligand of FGFR3 in *A*. *muricata*, *P*. *damicornis*, and *M*. *capricomis*, but not in *P*. *verrucosa*. These full-length transcriptomes could be exploited by other researchers to carry out phylogenetic studies as well as functional analyses. Applying controlled release of FGF8 produced on an industrial scale in the marine environment is a potential method to induce polyp survival, coral recovery, and growth, and should thus be further investigated.

Owing to this study, more knowledge regarding the FGF-FGFR binding patterns and mechanisms in reef-building corals has been gained. However, several questions remain unanswered. Although FGFs and FGFRs, which are potential budding reproduction proteins, have been identified in the four studied species, the specific mechanisms of the FGF signaling pathway in actual coral growth are still unclear. Further experiments on FGFR expression in the budding site are required to fully understand these mechanisms to enable its application to coral reef conservation and protection.

## Data Availability Statement

The datasets presented in this study can be found in online repositories. The names of the repository/repositories and accession number(s) can be found in the article/Supplementary Material.

## Author Contributions

ZG: experiment, writing and editing. ZL: reviewing. CH: supervision. XL: project approval. All authors contributed to the article and approved the submitted version.

## Conflict of Interest

The authors declare that the research was conducted in the absence of any commercial or financial relationships that could be construed as a potential conflict of interest.

## Publisher’s Note

All claims expressed in this article are solely those of the authors and do not necessarily represent those of their affiliated organizations, or those of the publisher, the editors and the reviewers. Any product that may be evaluated in this article, or claim that may be made by its manufacturer, is not guaranteed or endorsed by the publisher.
