# Supplementary material for: Binding Pattern Reconstructions of FGF-FGFR Budding-Inducing Signaling in Reef-Building Corals
Source: Front Physiol. 2022 Jan 4;12:759370. doi: 10.3389/fphys.2021.759370 (PMC8764167; doi:10.3389/fphys.2021.759370)
Supplement: Supplementary file 1 [file Data_Sheet_2.DOCX]

Supplementary Material

# Supplementary Data

>Pd_FGF8

MALGVNLLRVLTLLTLHSLLPDGLAEESGVNQKPEEKKVYDQHYLEQKAKSDTNSVAFRRFIQLYSRNSGRHVRINPDRSVDAIGEDGDKYAKLVIESVNFGRVRIKGSVSNFYLCVDKKGRLRARKRGKWKDNCEFTDRVADNAYTEFTSVRYNKSLIAFSRRGRPRPVLSTEKGGVKAVQFIERASNIKLLRGRKYWRKGGKLEGIDLYRKKQVEKIYVSMKKWREFKRWLKFNTRTASAKVNSTTTAATPLQPTAVQLSSRPTSFMKYSRDKGRT

>Pd_FGFR3

MALKSNALLVWLWITLFVGCQFKATAQPDHGKTCHELHRLCVRDVQFQNCTFSHLVPSISQEELFNISVRVNGLAKFTCLQTNNEASLATVPHIDWIKWKNTSSADKLDVDFGNFTSIVSNSKYNIKPDVKEHLHVSYLTIHNVTETDVGLYSCVVCNSYGRVYQSALLSLNTTLDPAVHTTSTPKTVDSRTLTTQSTSQSMATQRIKVIVGCIGAFVGVILLAVVVFFFKWRQKEPKNQGLPIILHEPDSPEEQIDGTPPLSVKFRNGLLIASTQRDSLLPLIHKRNSSYRSQLSSSASGGTVVTYADDLFEYPLDEKWEVPRETLFIKDLAGEGAFGYVAKAEAFQLPNTSTPCTVAVKMLKENATDAELSDLISEMETMKEIGSHKNIVNFLGACTVQGPLYLIVEYCPHGNLRDFLRDNRPSLTDINGKTTAQLTLRDLLSIAYQIARGMSYLSSKKCIHRDLAARNVLIAEDFVIKIADFGLSRNLGNTDYYRRTTHGRLPVKWLAIEALFDQQYTVKTDVWSFGIILWEIFTLGGTPYPGIPVERLFTILKNGYRMECPINCPDNIYKIMVKCWSENAKSRPEFFELTEQLDELLSSESSQEYIEVLAKSVDCLAEVEMGSGQTAESQNPQEANIIHENMAIKGSEGKDIPFSDTNLQLTS

>Pv_FGF8

MRSKLSATVKSDMLLFSMICWITFILGRGQEANIFKDHVRAERRRSDTRSLPFHRETQLYSRASRSHLRINGKHIDAMGRDGDKYAKLVIESDNFGRVRIRGALTNYYLCVKRNASLIGRRATKSKRCVFYEKYAENHYTEFLSAYNESWTIAVSKKGKMRPGHKARRGQRNAQFIERASKIIIQTKNVREALYHGLRDHIEQLLRAYRAREGGENKEKPRKMPHPGYSSGSWKRKAKIKRREKKMKLRSEKRLLKLLRKEQWRERRKEKF

>Pv_FGFR3

MTSTKPPQERVDQKFNLQFGSQIIIMIIFLQARSSEGVSPKEASRLNGSIVDHFERVGCFKDKKPKRALPELLHTFAVNWTDMNNSYSKIIRACAVNVSKAGFRYFGIQHYKECWSGVKGSVTYNKYGRNNNCDLLNGIGKSWSNFVYRFVEVNGSWSHWSPWQPCNSTCGGGYRSRVRTCTNPRPKWNGLDCNGSSISNGSCNVHSCADHEKSEPFSKRSSLPLVGLWLGISLVVLLCLSGILWITLRFSRKRRSNSQLVDPFEIVFDDISLCNLIGEGAFGKVYSAELNKQMETGKEGKSSVAKTNKQKLPKQKYRIVAVKMLRGGATEEQKEEFLEEIKLMKLIGYHRHVLNLLACCTNTVPMFLVTEFAKYGDLLNFLRKRREQIKQAIEEMEAAKLYQSRCCNLGLSTSEANLYDSTDDIDKYTTGLGSFCNKNGYGSNLKTDPHILYEDDTLRPADLLTFAWQIAQAMEFLSCKGFVHRDLAARNVLVSEGKMMKVADFGLSRDVYAEKVYHSTKTKKLPIKWMSPEAIHDQVFTTESDVWSFGILLWEMATIGGTPYPTISNQRILKALNSGYRMEKPQICSDEMYELMRQCWREKPSERPSFPFIREQLERMMLSRSPYLELTDASYTHVAYSETDSDEDTIQENTAL

>Mc_FGF8

MKLKFTTRCRRYLLIFSLNCLLSQSLGQQSQEKKYLDNHVKTERKRSDTRSLPFHRETQLYSRSSRSHLRIHGKKIDALGRDGDNYAKLVIESDNFGRVRIRGALTCYYLCIKRNATFIGRKATKSRRCVFYEKYAENHYTEFVSAYNESWTIAVSKKGNMRPGYKGRRGQRTVQFIERASKIIIQTKNVDESLYHGLRDHIEQLLQAYRAKEEDGDKDKPRKMPHPARYKQERQRKVKIKRKEKKMKRRSKNALLKLLRKERHKAEKRDSR

>Mc_FGFR3

MFLSALAFIPISLVFLCQARSEPCPVPEIRNKGDKIEVVSIGESKDAKLICVVKNTDNYKPTVSWEKDNKTLHPANHQRMRLKANRYLKIKSARKEDEGLYTCVAENTCGGRNTLSLRLFVEGPTLDPKRNSTPAAPEFTVPEEKRRRNLLAVPVGNSVKMDCSAFGFPRPTVTWYKDGVIYQARKGGSRLYISKLRNVVTIKDVVPSDSGLYTCNVSNAHGWINHSYRVDVHERVRAKPVILEMENVTVMEGENATLLCKALSDSMPHFQWLRWLAPPTNASGNISNPLYEVIKQNDQGGNSHLLVRHGETSKLEFHGVKLTLVNVTKKDEGKYTCIVGNAVGYTVEEAYIFVRELSEVALSQETSTSVSNNTSPLTDVNRFQEVGISAVPSSRKKIFIAIAVGFGFLTVAGVGLLLFCYRRKLKSSKATNYGIVYKAEAKETELQYRAHDPSLGGSSSSYCSTVPLIRNRSLRSRLGSNLTQVSEVEMPMDEKWEIDRENINMLGVLGEGAFGRVMKAEILGLPNMPFKFDVAVKMLKEDATDHEFADLVSEMETMKTIGKHKNIINLIGACTQGGPLHVVVEYAPNGNLRQFLRDRRPTREYTTTLTLADLVSFGYQVVRGMEYLSSKKCIHRDLAARNILVGEENTLKIADFGLARDVHQIDYYRKTTDGRLPVKWMALEALFDRVYTIQSDVWAFGILVWEIVTFGGSPYPSVPIENLFELLKFGYRMEKPVNCPDNMYEIMLRCWQDSPSQRPTFTELVKEFDAMLMSLSDKEYIDLEASQISSMEPQTPTSSEAPSTPRHSVATEHDTESDDRDNDNVFVDNSNDIWESRRHELPYHVRQPENALDITRSLFQSEGQIERNHARHKSSLGADENKKKSSFATFRSPIQSDV

>Am_FGF8

MALGLKLFRALVLLTLYLLSHGLAQASNLDQRPEEQKLFDDIYGNQKTKTDTKSIPFCRFIQLYSRNSGRHVRIKEDRSVDASGEDGDKYAKLIIESVSFGRVHIRGSVTNFYLCVDKRGRLKARARGKWKDNCVFTDHLADNAFTEFRSVKYNKTLIAFNRSGRPRQVNRPFRAGMKAFQFIERALNIRLYKGRRYKISNGRGSNRIDLYRRKHTYDKIFVSYKKWKEFRRWLKLSKKKVTEKKNKPLTAAPEPRVQATVQLVSNNTASITLL

>Am_FGFR3

MGKKRIVQSAWIFMNVFILKVIAQPDGPEGICHEEHGRCIRDSQFQNCTNNAHTPRISKKNLLNISITQNNLATFSCKQEILGAGLAGLQFDWIKWSNDISLYCSLDLDNGNFTVIDENSKFSIKPSAPDDIGNHWSYLVIHNATVHDSGLYSCVVCNQHGRDFSSAFLTVTTPSTPARAFTGSSSPSSSHHSPSFLSAKIILGSIGGIIAVILVAIAFLVWKRRRSKDQSLLTILTWKKAINDENISEHSIQVKQQTVELTGETVAVSQGDILAPQAQKRHSSYRSQLSSAGSLGTVITYADDLLDIPLDENWEIPRDSIDIKELAGEGAFGYVAKAKAFQLPNRVSTPCIVAVKMLKENASDVELADFISEMEMMKEIGSHKNIVNYLGACTVHGPLFLIVEYCSHGNLKDFLRNNRPSLLELSGDMEVSLTFRDLLSFAYQITKGMSYLSSKKCIHRDLAARNVLIAEDFVIKIADFGLSRNLGNTDYYRRTTHGRLPVKWLAIEALFDQQYTMKTDVQTNEKLLFLSSLGGSPYPGIPVEQLFSLLKTGYRMECPINCPTEMYEIMMQCWSDDARNRPSFDELYGKLDFMLSTETAMEYVAILAQSVDCLAEPEAESEEPCPRTKQQTNRINTTI

# Supplementary Figures and Tables

## Supplementary Figures


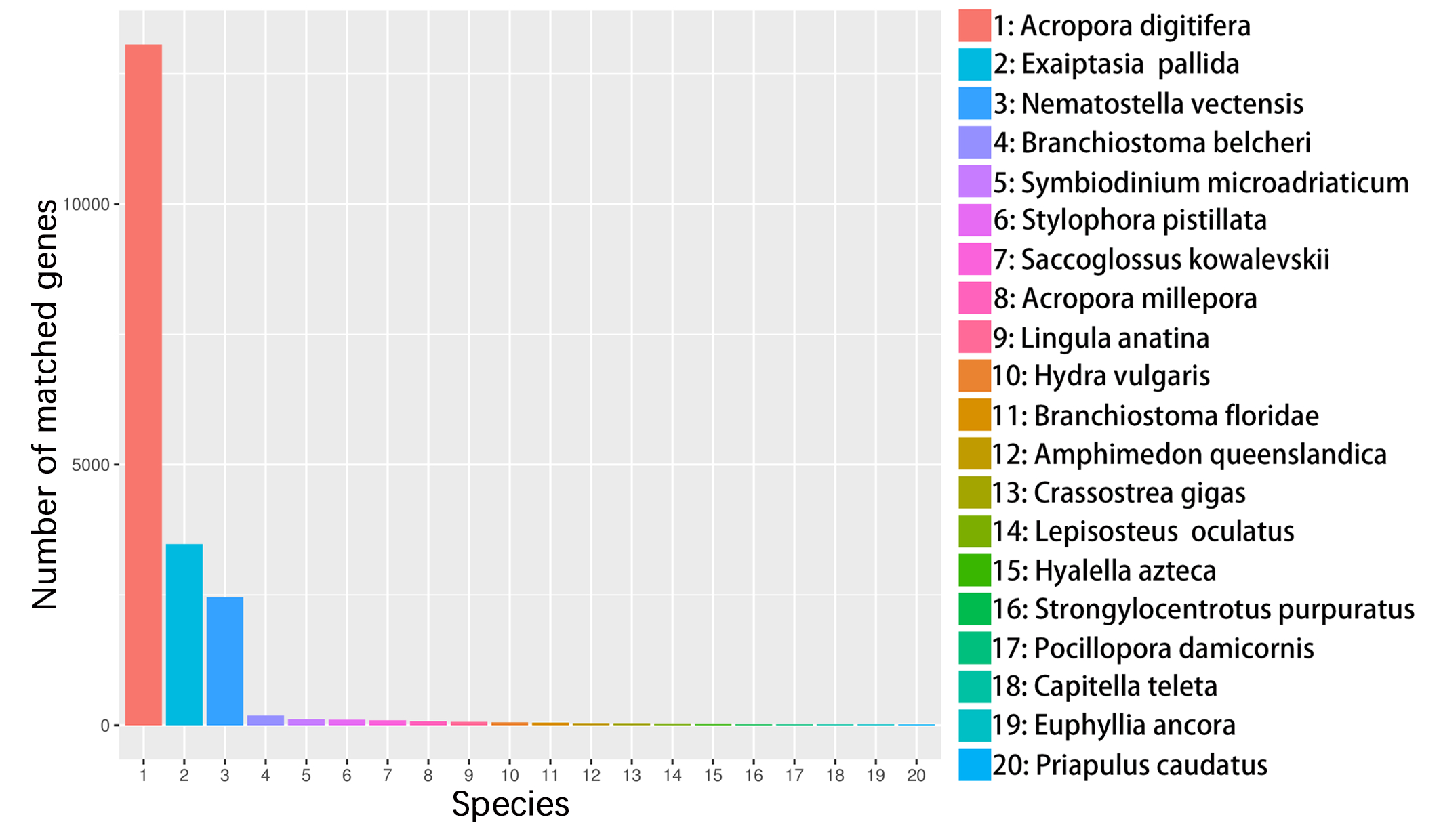


**Supplementary Figure 1. Annotation statistical results of *P*. *damicornis* full-length transcriptome according to the NR database.** The horizontal axis is the species ID and the vertical axis is unigene number. The top three species with highest number of annotated unigenes are *A*. *digitifera*, *Exaiptasia* *pallida*, and *Nematostella vectensis*, together comprising 84.7% of the unigenes.


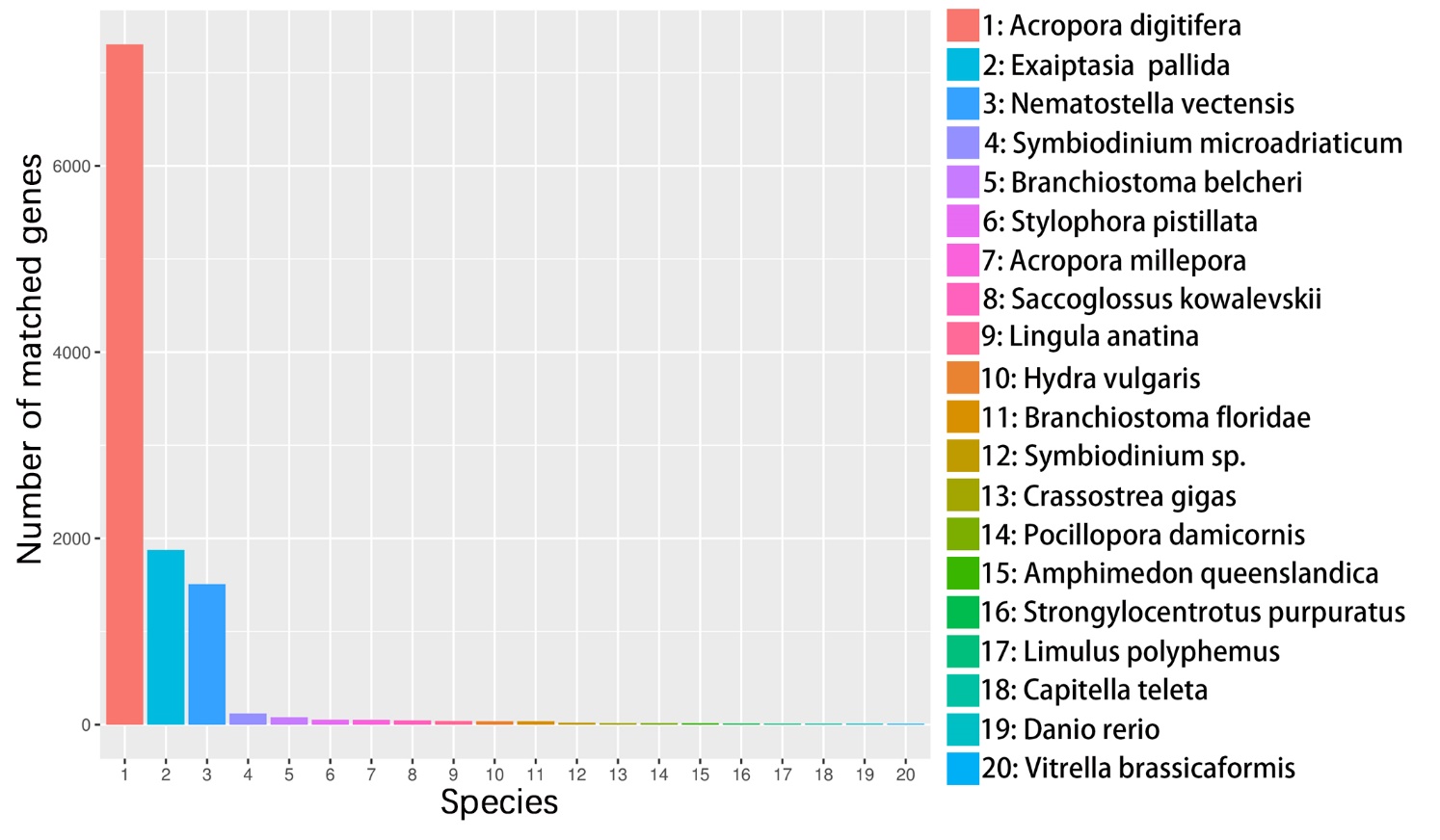


**Supplementary Figure 2.** **Annotation of *P*. *verrucosa* full-length transcriptome with to NR database.** Horizontal axis is species ID and vertical axis is unigene number. The top three species with highest number of annotated unigenes are *A*. *digitifera*, *Exaiptasia pallida*, and *Nematostella vectensis*, which together account for 81.1% of unigenes.


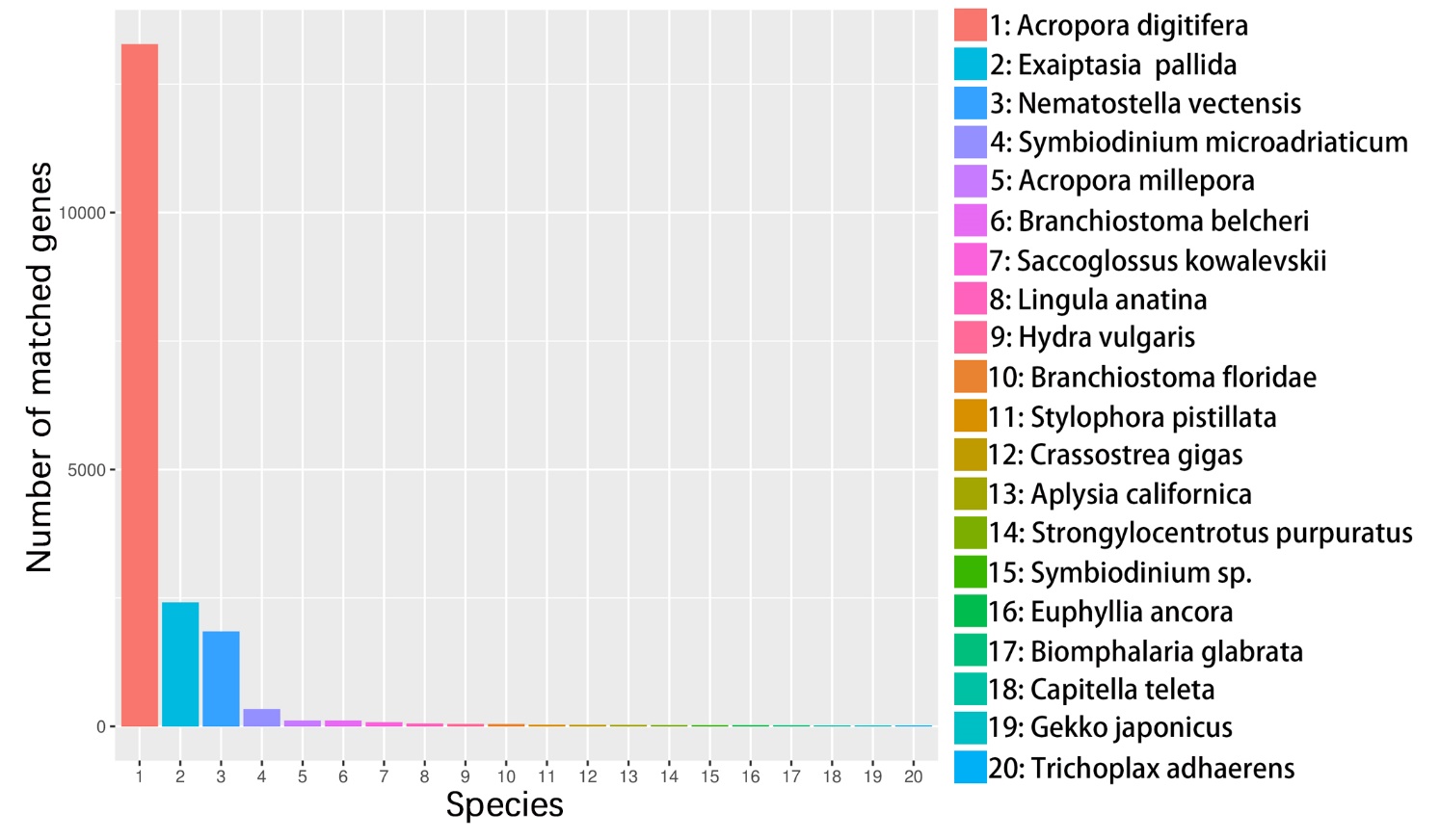


**Supplementary Figure 3. Annotation of *M*. *capricomis* full-length transcriptome with NR database.** Horizontal axis is species ID and vertical axise is unigene number. The top three species with the highest number of annotated unigenes are *A*. *digitifera*, *Exaiptasia pallida*, and *Nematostella vectensis*, which together account for 86.6% of unigenes.


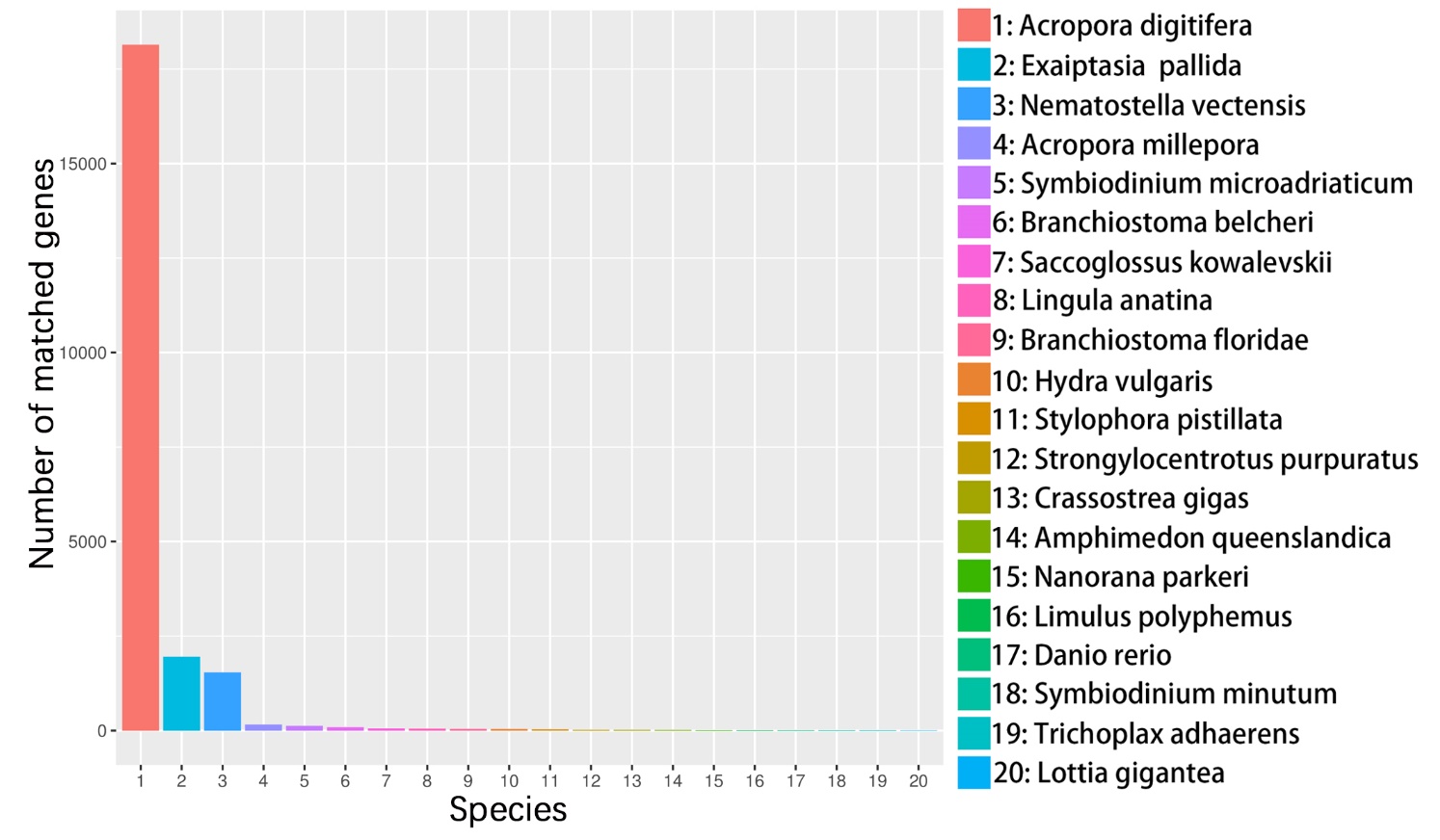


**Supplementary Figure 4. Annotation of *A. muricata* full-length transcriptome with NR database.** Horizontal axis is species ID and vertical axise is unigene number. The top three species with the highest number of annotated unigenes are *A*. *digitifera*, *Exaiptasia pallida*, and *Nematostella vectensis*, which together account for 92.0% of unigenes.


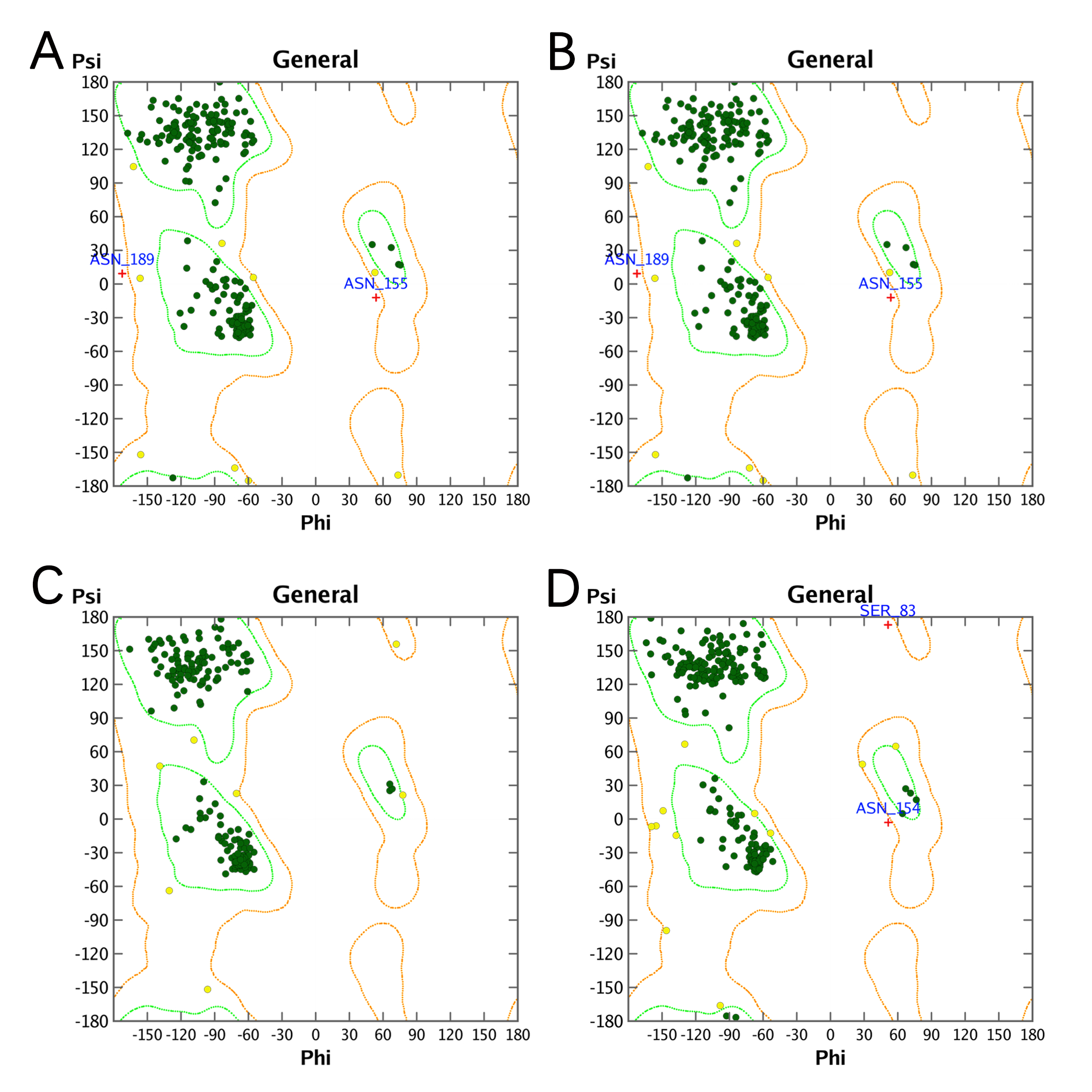


**Supplementary Figure 5. Ramachandran plots of coral FGF8 proteins.** (A)-(D) are the Ramachandran plots of FGF8 proteins for *P*. *damicornis*, *P*. *verrucosa*, *M*. *capricomis* and *A*. *muricata*. In a Ramachandran plot, every dot represents an amino acid residue. Dark green dots represent the residues in favored regions, yellow dots represent the residues in allowed regions, and red crosses represents residues in irrational regions. These plots show that over 99% of proteins in our reconstructions are in favored regions, demonstrating that the constructed homology models are realistic.


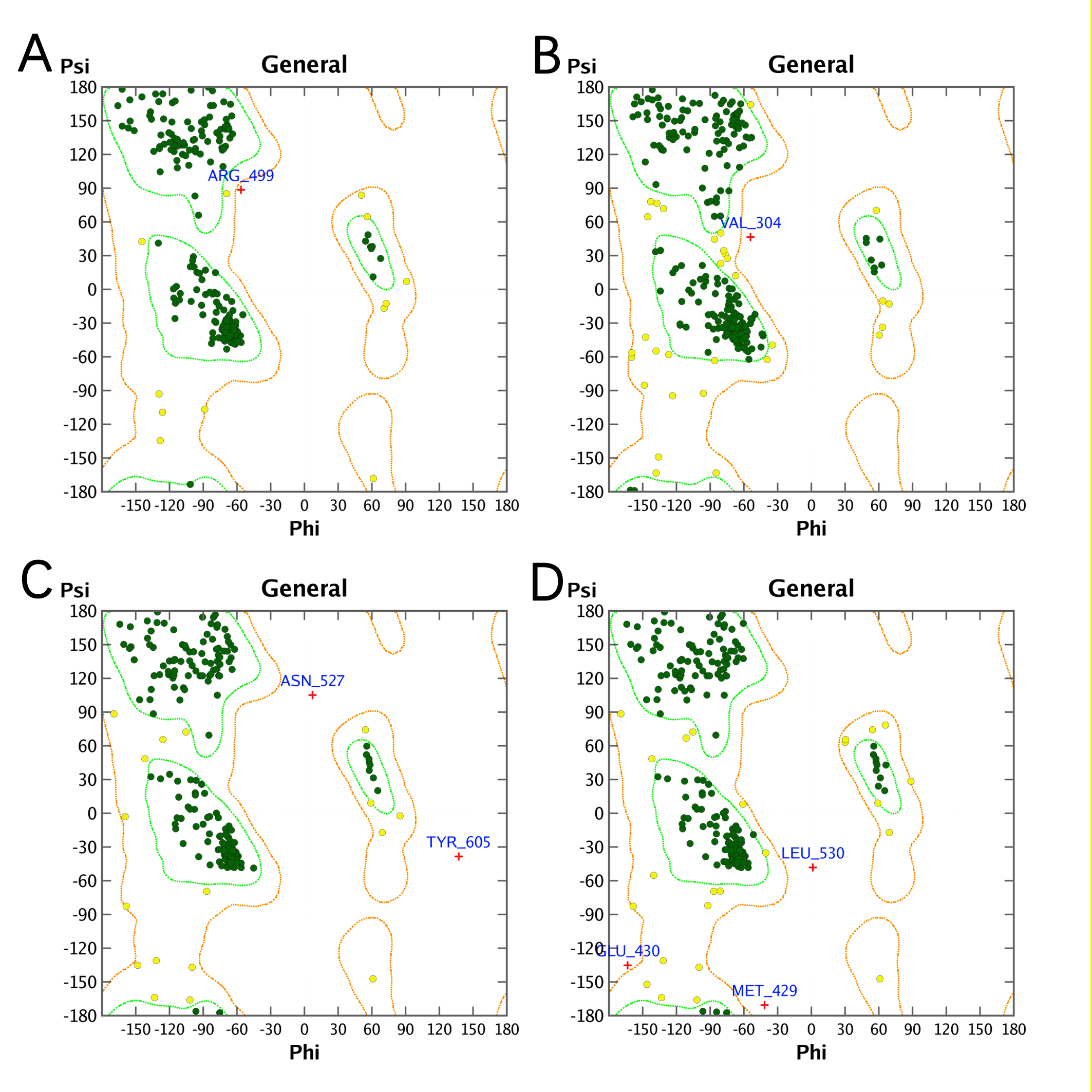


**Supplementary Figure 6. Ramachandran plots of coral FGFR3 proteins.** Ramachandran plots of FGFR3 proteins for *P*. *damicornis*, *P*. *verrucosa*, *M*. *capricomis* and *A*. *muricata* are shown in (A)-(D). As in Supplementary Figure 5, every dot represents an amino acid residue. Dark green dots represent tresidues in favored regions, yellow dots represent residues in allowed regions, and red crosses represent residues in irrational regions. These plots show that over 99% of amino acids are in favored regions, demonstrating that the constructed models are realistic.


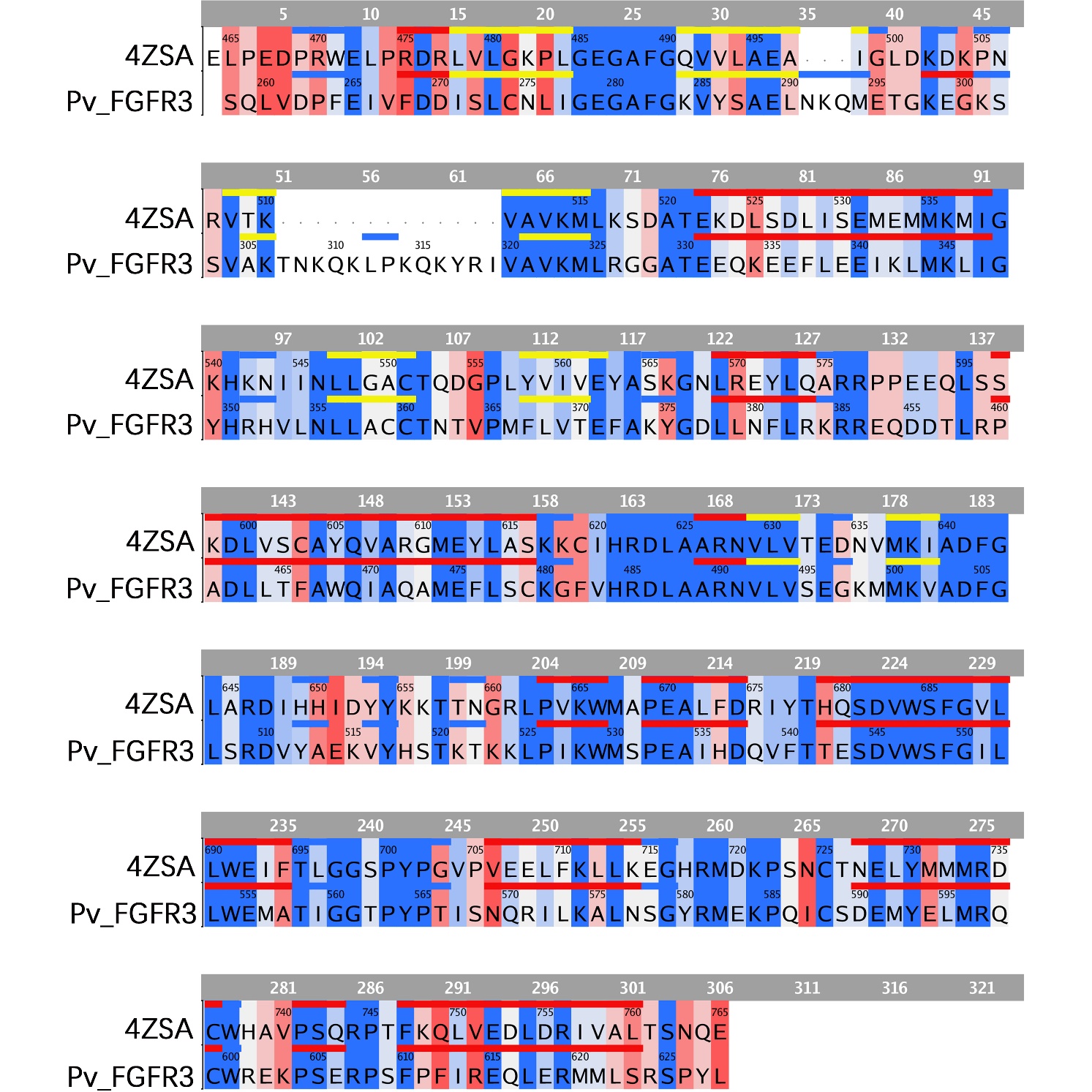


**Supplementary Figure 7. Sequence comparison between FGFR3 in *P*. *verrucosa* with its template.** The same or similar residues are highlighted in blue and dissimilar ones are highlighted in red, with darker blue indicating more similar residues and darker red indicating more dissimilar residues. The sequences corresponding to alpha helixes and beta strands are marked with red and yellow lines, respectively.


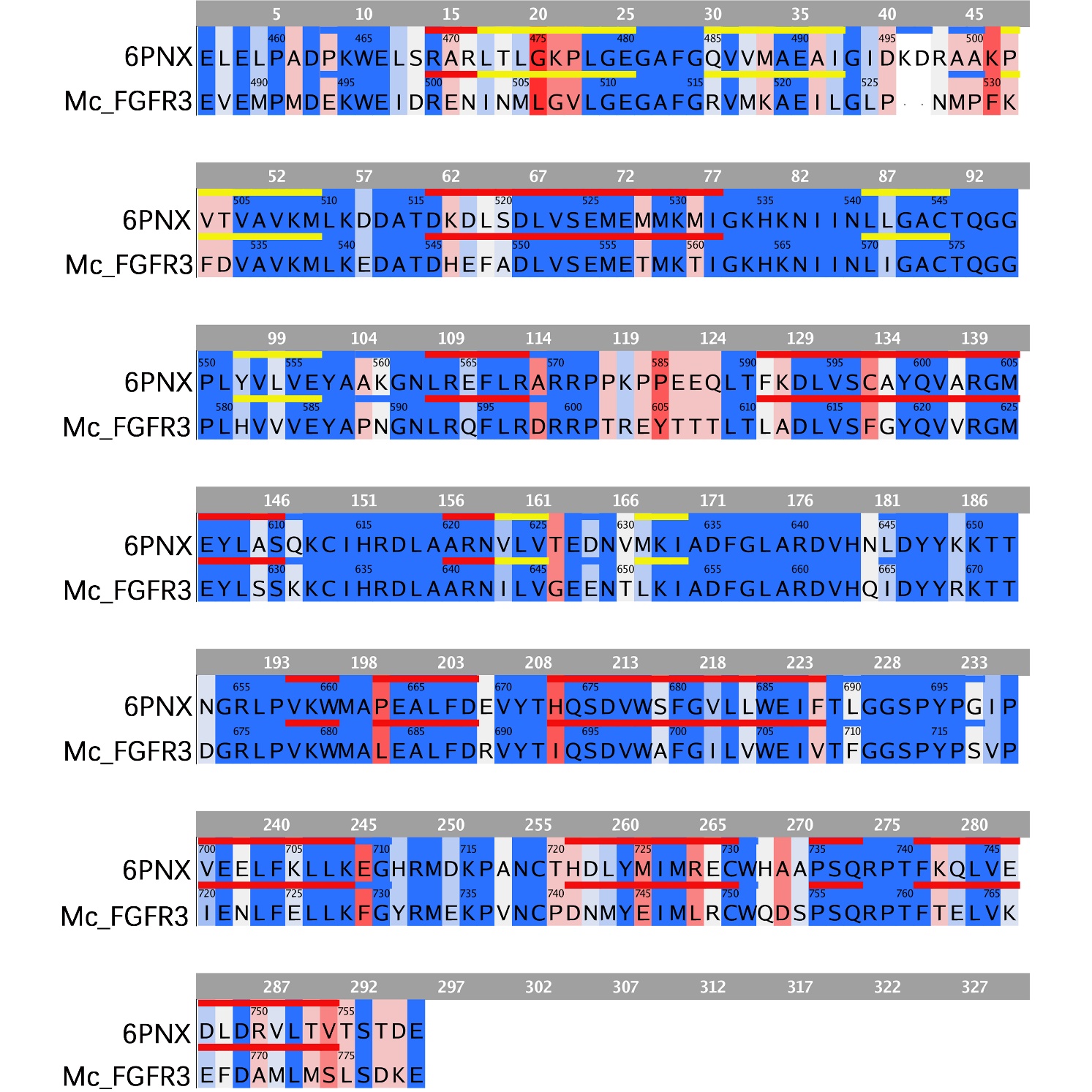


**Supplementary Figure 8. Sequence comparison between FGFR3 in *M*. *capricornis* with its template.** The same or similar residues are highlighted in blue and dissimilar ones are highlighted in red, with darker blue indicating more similar residues and darker red indicating more dissimilar residues. The sequences corresponding to alpha helixes and beta strands are marked with red and yellow lines, respectively.


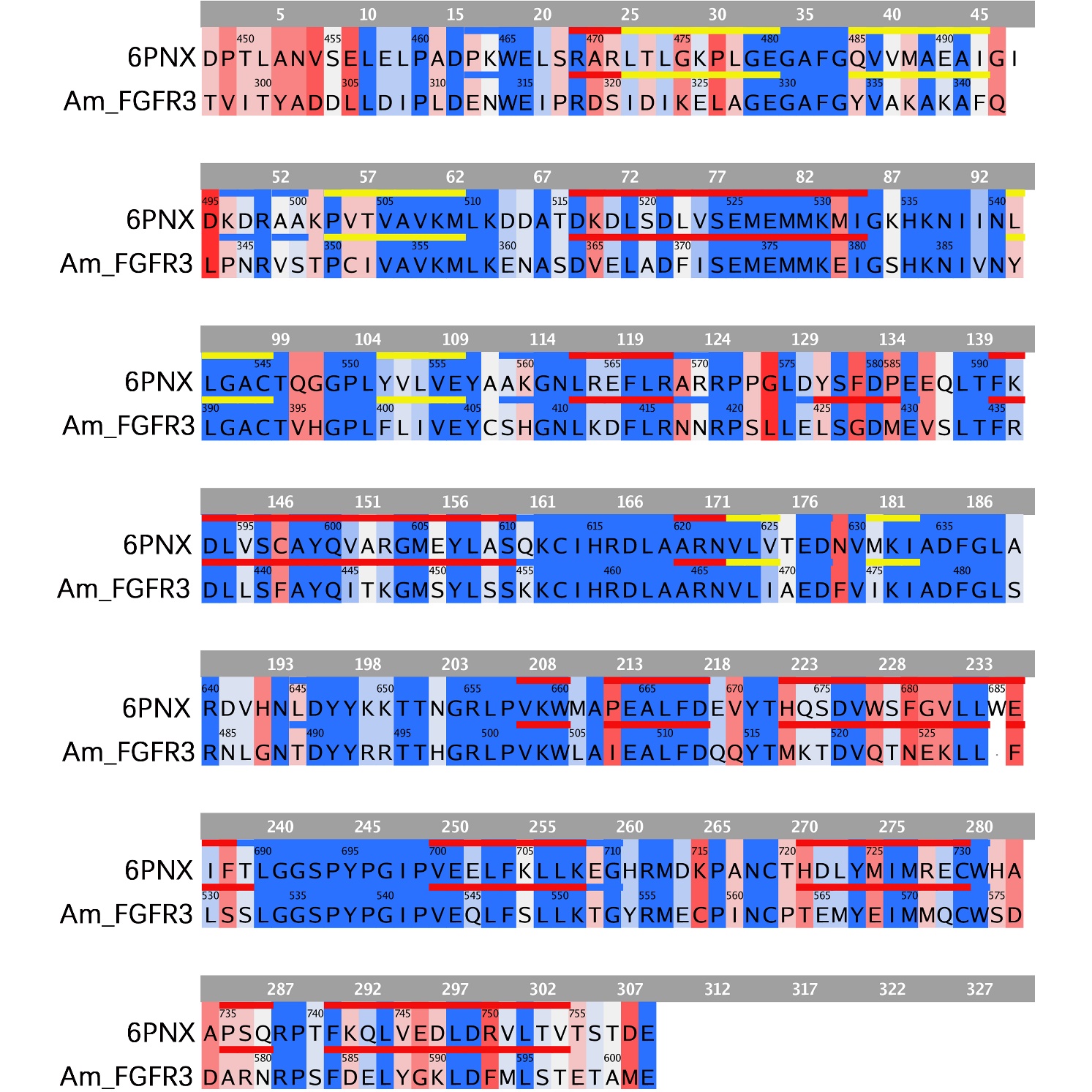


**Supplementary Figure 9. Sequence comparison between FGFR3 in *A*. *muricata* with its template.** The same or similar residues are highlighted in blue and dissimilar ones are highlighted in red, with darker blue indicating more similar residues and darker red indicating more dissimilar residues. The sequences corresponding to alpha helixes and beta strands are marked with red and yellow lines, respectively.


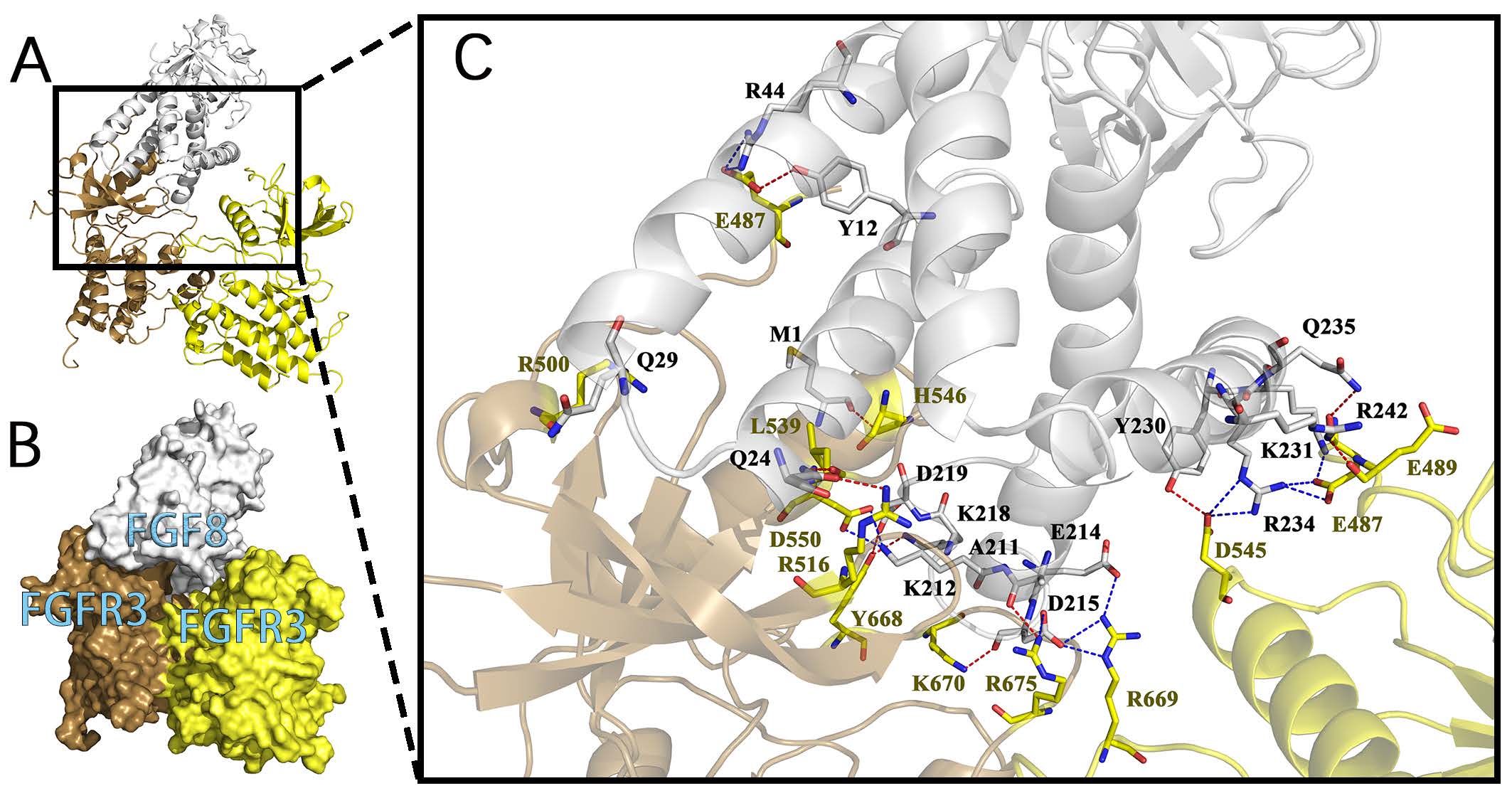


**Supplementary Figure 10. FGF8-FGFR3 binding pattern in *M*. *capricomis*.** (A) The interaction between Mc_FGF8 and Mc_FGFR3. (B) Surface-binding model of Mc_FGF8 and Mc_FGFR3. Mc_FGFR3 chain A is yellow, Mc_FGFR3 chain B is brown and Mc_FGF8 is white. (C) Detail of interaction between Mc_FGF8 and Mc_FGFR3. The residues in Mc_FGFR3 are yellow, and in Mc_FGF8 they are white. The red dashes represent hydrogen bond interactions and the blue dashes represent salt bridges.


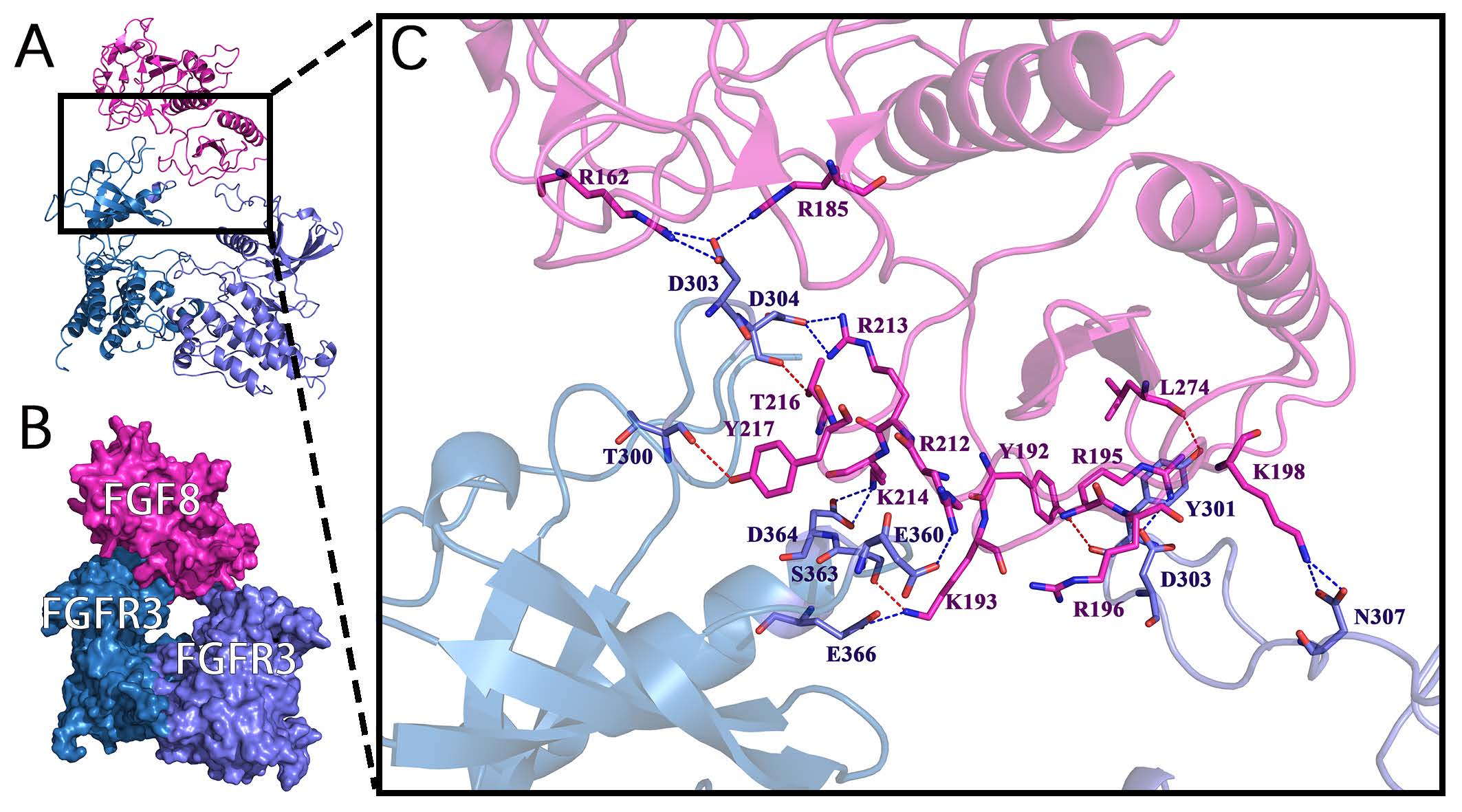


**Supplementary Figure 11. FGF8-FGFR3 binding pattern docked by ClusPro in *A*. *muricata*.** (A) The interaction between Am_FGF8 and Am_FGFR3. (B) Surface-binding model of Am_FGF8 and Am_FGFR3. Am_FGFR3 chain A is colored with purple, Am_FGFR3 chain B is colored with blue and Am_FGF8 is magenta. (C) Detail of interaction between Am_FGF8 and Am_FGFR3. The residues in Am_FGFR3 are purple, and in Am_FGF8 they are magenta. The red dashes represent hydrogen bond interactions and the blue dashes represent salt bridges.

## Supplementary Tables

**Supplementary Table 1. List of contacts between Pd_FGFR3 and Pd_FGF8 in docking.**

| Chain 1 | Residue | Chain 2 | Residue | Interaction type |
| --- | --- | --- | --- | --- |
| Pd_FGFR3.A | Phe312.O | Pd_FGF8.C | Arg231.NH2 | Hydrogen bond interaction |
| Pd_FGFR3.A | Glu313.O | Pd_FGF8.C | Arg231.NH2 | Hydrogen bond interaction |
| Pd_FGFR3.A | Glu318.O | Pd_FGF8.C | Arg163.NH1 | Hydrogen bond interaction |
| Pd_FGFR3.A | Lys319.O | Pd_FGF8.C | Arg71.NH1 | Hydrogen bond interaction |
| Pd_FGFR3.A | Glu321.O | Pd_FGF8.C | Arg163.NH1/NH2 | Hydrogen bond interaction |
| Pd_FGFR3.A | Gln348.OE1 | Pd_FGF8.C | Arg168.NH1/NH2 | Hydrogen bond interaction |
| Pd_FGFR3.A | Thr352.OG1 | Pd_FGF8.C | Arg168.NH1 | Hydrogen bond interaction |
| Pd_FGFR3.A | Asp369.OD1 | Pd_FGF8.C | Ser262.N | Hydrogen bond interaction |
| Pd_FGFR3.A | Gln401.OE1 | Pd_FGF8.C | Arg67.NH2 | Hydrogen bond interaction |
| Pd_FGFR3.A | Asp310.OD1/OD2 | Pd_FGF8.C | Arg277.NH1/NH2 | Salt bridge |
| Pd_FGFR3.A | Asp317.OD1/OD2 | Pd_FGF8.C | Arg227.NH1/NH2 | Salt bridge |
| Pd_FGFR3.A | Glu318.OE1/OE2 | Pd_FGF8.C | Arg186.NH1/NH2 | Salt bridge |
| Pd_FGFR3.A | Lys319.NZ | Pd_FGF8.C | Asp87.OD1/OD2 | Salt bridge |
| Pd_FGFR3.A | Glu321.OE2 | Pd_FGF8.C | Arg67.NH2 | Salt bridge |
| Pd_FGFR3.A | Glu380.OE1 | Pd_FGF8.C | Lys224.NZ | Salt bridge |
| Pd_FGFR3.A | Glu384.OE1/OE2 | Pd_FGF8.C | Lys224.NZ | Salt bridge |
| Pd_FGFR3.B | Lys364.O | Pd_FGF8.C | Lys201.NZ | Hydrogen bond interaction |
| Pd_FGFR3.B | Glu365.O | Pd_FGF8.C | Glu206.N | Hydrogen bond interaction |
| Pd_FGFR3.B | Glu371.OE1 | Pd_FGF8.C | Ser267.OG | Hydrogen bond interaction |
| Pd_FGFR3.B | Glu335.OE1/OE2 | Pd_FGF8.C | Arg264.NH1/NH2 | Salt bridge |

**Supplementary Table 2. List of contacts between Pv_FGFR3 and Pv_FGF8 in docking.**

| Chain 1 | Residue | Chain 2 | Residue | Interaction type |
| --- | --- | --- | --- | --- |
| Pv_FGFR3.A | Gln259.OE1 | Pv_FGF8 | Arg62.NH2 | Hydrogen bond interaction |
| Pv_FGFR3.A | Gln259.NE2 | Pv_FGF8 | Asp81.OD2 | Hydrogen bond interaction |
| Pv_FGFR3.A | Asp262.OD1/OD2 | Pv_FGF8 | Arg242.NH2/NE | Salt bridge |
| Pv_FGFR3.A | Asp262.OD1/OD2 | Pv_FGF8 | Arg249.NH1/NH2 | Salt bridge |
| Pv_FGFR3.A | Pro263.O | Pv_FGF8 | Arg28.NH2 | Hydrogen bond interaction |
| Pv_FGFR3.A | Glu299.OE1/OE2 | Pv_FGF8 | Lys238.NZ | Salt bridge |
| Pv_FGFR3.A | Glu331.OE2 | Pv_FGF8 | Arg65.NH1/NH2 | Salt bridge |
| Pv_FGFR3.A | Glu332.OE2 | Pv_FGF8 | Lys157.NZ | Salt bridge |
| Pv_FGFR3.A | Glu335.OE2 | Pv_FGF8 | Arg65.NH2/NE | Salt bridge |
| Pv_FGFR3.A | Glu336.OE1/OE2 | Pv_FGF8 | Lys156.NZ | Salt bridge |
| Pv_FGFR3.A | Glu336.OE2 | Pv_FGF8 | Arg171.NH1 | Salt bridge |
| Pv_FGFR3.A | Glu339.OE2 | Pv_FGF8 | Tyr139.OH | Hydrogen bond interaction |
| Pv_FGFR3.A | Lys342.NZ | Pv_FGF8 | Arg62.O | Hydrogen bond interaction |
| Pv_FGFR3.A | Arg485.NH1/NH2 | Pv_FGF8 | Arg168.O | Hydrogen bond interaction |
| Pv_FGFR3.A | Ser508.OG | Pv_FGF8 | Tyr139.OH | Hydrogen bond interaction |
| Pv_FGFR3.A | Asp510.OD1/OD2 | Pv_FGF8 | Lys156.NZ | Salt bridge |
| Pv_FGFR3.A | Tyr512.OH | Pv_FGF8 | Lys165.NZ | Hydrogen bond interaction |
| Pv_FGFR3.A | Asp537.O | Pv_FGF8 | Lys165.NZ | Hydrogen bond interaction |
| Pv_FGFR3.A | Gln538.O | Pv_FGF8 | Lys165.NZ | Hydrogen bond interaction |
| Pv_FGFR3.A | Gln538.OE1 | Pv_FGF8 | Arg167.NH1 | Hydrogen bond interaction |
| Pv_FGFR3.A | Gln259.OE1 | Pv_FGF8 | Arg62.NH2 | Hydrogen bond interaction |

**Supplementary Table 3. List of contacts between Mc_FGFR3 and Mc_FGF8 in docking.**

| Chain 1 | Residue | Chain 2 | Residue | Interaction type |
| --- | --- | --- | --- | --- |
| Mc_FGFR3.A | Glu487.O | Mc_FGF8 | Gln235.NE2 | Hydrogen bond interaction |
| Mc_FGFR3.A | Glu489.O | Mc_FGF8 | Arg242.NH2 | Hydrogen bond interaction |
| Mc_FGFR3.A | Asp545.OD1 | Mc_FGF8 | Tyr230.OH | Hydrogen bond interaction |
| Mc_FGFR3.A | Glu487.OE2 | Mc_FGF8 | Lys231.NZ | Salt bridge |
| Mc_FGFR3.A | Glu487.OE1/OE2 | Mc_FGF8 | Arg234.NH1 | Salt bridge |
| Mc_FGFR3.A | Asp545.OD1 | Mc_FGF8 | Arg234.NH2/NE | Salt bridge |
| Mc_FGFR3.A | Arg669.NH2 | Mc_FGF8 | Glu214.OE1 | Salt bridge |
| Mc_FGFR3.A | Arg669.NH2/NE | Mc_FGF8 | Asp215.OD1 | Salt bridge |
| Mc_FGFR3.B | Hsd546.NE2 | Mc_FGF8 | Met1.O | Hydrogen bond interaction |
| Mc_FGFR3.B | Glu487.OE1 | Mc_FGF8 | Tyr12.OH | Hydrogen bond interaction |
| Mc_FGFR3.B | Arg516.NH1 | Mc_FGF8 | Gln24.OE1 | Hydrogen bond interaction |
| Mc_FGFR3.B | Leu539.O | Mc_FGF8 | Gln24.NE2 | Hydrogen bond interaction |
| Mc_FGFR3.B | Arg500.O | Mc_FGF8 | Gln29.NE2 | Hydrogen bond interaction |
| Mc_FGFR3.B | Arg675.NH1/NH2 | Mc_FGF8 | Ala211.O | Hydrogen bond interaction |
| Mc_FGFR3.B | Lys670.NZ | Mc_FGF8 | Asp215.O | Hydrogen bond interaction |
| Mc_FGFR3.B | Tyr668.OH | Mc_FGF8 | Lys218.NZ | Hydrogen bond interaction |
| Mc_FGFR3.B | Tyr668.OH | Mc_FGF8 | Asp219.OD2 | Hydrogen bond interaction |
| Mc_FGFR3.B | Glu487.OE2 | Mc_FGF8 | Arg44.NH1/NH2 | Salt bridge |
| Mc_FGFR3.B | Asp550.OD1/OD2 | Mc_FGF8 | Lys212.NZ | Salt bridge |
| Mc_FGFR3.B | Arg675.NH2 | Mc_FGF8 | Asp215.OD1 | Salt bridge |

**Supplementary Table 4. List of contacts between Am_FGFR3 and Am_FGF8 in docking.**

| Chain 1 | Residue | Chain 2 | Residue | Interaction type |
| --- | --- | --- | --- | --- |
| Am_FGFR3.A | Tyr301.O | Am_FGF8 | Tyr192.OH | Hydrogen bond interaction |
| Am_FGFR3.A | Tyr301.OH | Am_FGF8 | Leu274.O | Hydrogen bond interaction |
| Am_FGFR3.A | Asp303.OD2 | Am_FGF8 | Arg196.N | Hydrogen bond interaction |
| Am_FGFR3.A | Asp303.OD2 | Am_FGF8 | Arg195.NH1 | Salt bridge |
| Am_FGFR3.A | Asp307.OD1/OD2 | Am_FGF8 | Lys198.NZ | Salt bridge |
| Am_FGFR3.B | Thr300.O | Am_FGF8 | Tyr217.OH | Hydrogen bond interaction |
| Am_FGFR3.B | Asp304.O | Am_FGF8 | Thr216.OG1 | Hydrogen bond interaction |
| Am_FGFR3.B | Ser363.OG | Am_FGF8 | Lys193.NZ | Hydrogen bond interaction |
| Am_FGFR3.B | Asp303.OD1/OD2 | Am_FGF8 | Arg162.NH1/NH2 | Salt bridge |
| Am_FGFR3.B | Asp303.OD1 | Am_FGF8 | Arg185.NH1/NH2 | Salt bridge |
| Am_FGFR3.B | Asp304.OD2 | Am_FGF8 | Arg213.NH1/NH2 | Salt bridge |
| Am_FGFR3.B | Glu360.OE1 | Am_FGF8 | Arg212.NH1 | Salt bridge |
| Am_FGFR3.B | Asp364.OD1/OD2 | Am_FGF8 | Lys214.NZ | Salt bridge |
| Am_FGFR3.B | Glu366.OE1 | Am_FGF8 | Lys193.NZ | Salt bridge |
